# Genome-wide association and functional genomic analyses for body conformation traits in North American Holstein cattle

**DOI:** 10.3389/fgene.2024.1478788

**Published:** 2024-10-24

**Authors:** Luis Paulo B. Sousa Junior, Luis Fernando B. Pinto, Valdecy A. R. Cruz, Gerson A. Oliveira Junior, Hinayah R. Oliveira, Tatiane S. Chud, Victor B. Pedrosa, Filippo Miglior, Flávio S. Schenkel, Luiz F. Brito

**Affiliations:** ^1^ Department of Animal Sciences, Federal University of Bahia, Salvador, Brazil; ^2^ Department of Animal Sciences, Purdue University, West Lafayette, IN, United States; ^3^ Centre for Genetic Improvement of Livestock (CGIL), Department of Animal Biosciences, University of Guelph, Guelph, ON, Canada; ^4^ PEAK, Madison, WI, United States; ^5^ Lactanet Canada, Guelph, ON, Canada

**Keywords:** dairy cattle, high density genotypes, GWAS, type traits, imputation

## Abstract

Body conformation traits are directly associated with longevity, fertility, health, and workability in dairy cows and have been under direct genetic selection for many decades in various countries worldwide. The main objectives of this study were to perform genome-wide association studies and functional enrichment analyses for fourteen body conformation traits using imputed high-density single nucleotide polymorphism (SNP) genotypes. The traits analyzed include body condition score (BCS), body depth (BD), bone quality (BQ), chest width (CW), dairy capacity (DC), foot angle (FAN), front legs view (FLV), heel depth (HDe), height at front end (HFE), locomotion (LOC), rear legs rear view (RLRV), rear legs side view (RLSV), stature (ST), and a composite feet and legs score index (FL) of Holstein cows scored in Canada. De-regressed estimated breeding values from a dataset of 39,135 North American Holstein animals were used as pseudo-phenotypes in the genome-wide association analyses. A mixed linear model was used to estimate the SNP effects, which ranged from 239,533 to 242,747 markers depending on the trait analyzed. Genes and quantitative trait loci (QTL) located up to 100 Kb upstream or downstream of the significant SNPs previously cited in the Animal QTLdb were detected, and functional enrichment analyses were performed for the candidate genes identified for each trait. A total of 20, 60, 13, 17, 27, 8, 7, 19, 4, 10, 13, 15, 7, and 13 genome-wide statistically significant SNPs for Bonferroni correction based on independent chromosomal segments were identified for BCS, BD, BQ, CW, DC, FAN, FLV, HDe, HFE, LOC, RLRV, RLSV, ST, and FL, respectively. The significant SNPs were located across the whole genome, except on chromosomes BTA24, BTA27, and BTA29. Four markers (for BCS, BD, HDe, and RLRV) were statistically significant when considering a much stricter threshold for the Bonferroni correction for multiple tests. Moreover, the genomic regions identified overlap with various QTL previously reported for the trait groups of exterior, health, meat and carcass, milk, production, and reproduction. The functional enrichment analyses revealed 27 significant gene ontology terms. These enriched genomic regions harbor various candidate genes previously reported as linked to bone development, metabolism, as well as infectious and immunological diseases.

## 1 Introduction

Body conformation traits are economically important as they are associated with longevity, fertility, health, and workability in dairy cattle (Miglior et al., 2017; Oliveira Junior et al., 2021). Several dairy cattle breed organizations aim at selecting animals with optimal conformation traits, while improving production and other economically important traits, to maximize profitability (Fang and Pausch, 2019; Alcantara et al., 2022; Khmelnychyi et al., 2022). The Canadian classification system comprises 30 different linear type traits and defective characteristics, which are combined into trait groups (scorecards), i.e., including Mammary System, Feet and Legs, Dairy Strength, and Rump (Canada, 2023a). Moreover, an overall conformation score is calculated based on the importance of each trait group (Alcantara et al., 2022; Canada, 2023b). Body conformation traits in Holstein cattle are heritable, with heritability estimates ranging from 0.05 to 0.46 (Lactanet, 2021; Oliveira Junior et al., 2021).

An approach to detect significant associations between genetic and phenotypic variants is through genome-wide association studies (GWAS) that enables the estimation of the effects of each marker and the identification of the best candidates to discriminate the underlying biology of traits with polygenic traits (Sahana et al., 2023). Many GWAS have been reported for body conformation traits in Holstein cattle. For instance (Čítek et al., 2022), analyzed 25 body conformation traits, including angularity (ANG), body condition score (BCS), body depth (BD), bone quality (BQ), foot angle (FAN), chest width (CW), locomotion (LOC), rear legs rear view (RLRV), rear legs side view (RLSV), stature (ST), and composite feet and leg score (FL) and reported two candidate genes (CAMK2D, RANBP17) for BD and ANG in Czech Holsteins. In Chinese Holstein cattle, 105 genes within 200 kb up/downstream of the significant SNPs for heel depth (HDe), BQ, RLRV, and RLSV have also been reported (Abdalla et al., 2021). Moreover, another study reported 24 SNPs associated with 24 conformation traits in Korean Holstein cattle, including the RYBP gene associated with height at front end (HFE) (Haque et al., 2023; Ma et al., 2023) also performed a GWAS for monthly-recorded body weight (BW), hip height (HH), body length (BL), and chest girth (CG), followed by a multi-trait meta-analysis to detect pleiotropic markers in Chinese Holstein cattle. The authors reported 170 SNPs associated with the studied traits, including 17 SNPs with pleiotropic effects across body conformation traits, and various important candidate genes such as HMGA2, HNF4G, MED13L, BHLHE40, FRZB, DMP1, TRIB3, and GATAD2A.

Most GWAS for conformation traits in Holstein cattle, such as those mentioned previously, were performed using medium-density (MD; i.e., ∼50 K) SNP panels. One procedure for using more markers at lower costs is through genotype imputation, which adds missing genotypes based on a reference population (Calus et al., 2014). Genotype imputation from MD to high-density (HD) SNP panels can enable more accurate identification of quantitative trait loci (QTL) (Abo-Ismail et al., 2017; VanRaden et al., 2017). Thus, this study aimed at performing GWAS univariate analyses for 14 body conformation traits split into four groups: composite feet and legs score index (FL), foot score traits (FAN and HDe), dairy strength score traits (DC, BCS, BD, CW, HFE, and ST), and mobility score traits (BQ, LOC, FLV, RLRV, and RLSV) in North American Holstein cattle using imputed HD SNP data.

## 2 Materials and methods

### 2.1 Ethics statement

Phenotypic, pedigree, and genomic information were provided by Lactanet (www.lactanet.ca; Guelph, ON, Canada). Therefore, no animal experiments were carried out and the approval of the animal care committee was not needed.

### 2.2 Animals and phenotypes

The trait names, abbreviations, definitions, and heritability estimates are presented in Table 1. Between 15,269 and 24,893 Holstein cattle with de-regressed estimated breeding values (dEBVs) for BCS, BD, BQ, CW, DC, FL, FAN, FLV, HDe, HFE, LOC, RLRV, RLSV, and ST were included in this study (Table 2). The dEBVs were calculated according to VanRaden et al. (2009), and only dEBVs with reliability higher than 0.30 were kept for further analyses. Thirteen traits were measured individually on a 1–9 score system while the composite feet and legs score index (FL) was calculated as: $0.09\left(\text{FAN}\right)+0.22\left(\text{HDe}\right)+0.05\left(\text{BQ}\right)+0.31\left(\text{RLRV}\right)+0.19\left(\text{RLSV}\right)+0.14\;\left(\text{Thurl Placement}\right)$ (Canada, 2023a).

**TABLE 1:** Trait, abbreviations names, their short definitions, and heritability estimated.

Trait	Abbreviation	Short definition	Heritability^a^
Composite feet and legs score index			
Feet and legs	FL	Score of feet and legs that form a composite index	0.13
Foot Score Traits			
Foot angle	FAN	Angle of hairline at the hoof from 1 (low) to 9 (steep)	0.07
Heel depth	HDe	Depth of the heel on the outside claw from 1 (shallow) to 9 (deep)	0.08
Dairy Strength Score Traits			
Body condition score	BCS	Amount of fat deposition in the tailhead, loin and pelvic region	0.21
Body depth	BD	Depth of the body at the rear rib from 1 (shallow) to 9 (deep)	0.31
Dairy capacity	DC	Angle, openness and spring of ribs from 1 (nonangular) to 9 (angular)	0.18
Chest width	CW	Width at the chest floor from 1 (narrow) to 9 (wide)	0.20
Height at front end	HFE	Difference in height at the withers compared with the back of the animal from 1 (low) to 9 (high)	0.23
Stature	ST	Height at rump from 1 (short) to 9 (tall)	0.46
Mobility Score Traits			
Bone quality	BQ	Flatness of bone from 1 (coarse) to 9 (flat)	0.26
Front leg view	FLV	Square and straightness of front legs from 1 (knock-kneed) to 9 (bow-legged)	0.11
Locomotion	LOC	Straightness and length of strides from 1 (lame animals) to 9 (fluid)	0.05
Rear leg rear view	RLRV	Turn of the hock when viewed from rear from 1 (hocked-in) to 9 (straight)	0.20
Rear leg side view	RLSV	Degree of curvature of rear leg at the hock from 1 (straight) to 9 (curved)	0.11

^a^ (Lactanet, 2021).

**TABLE 2:** Descriptive statistics of the pseudo-phenotypes used for the genome-wide association analyses of conformation traits in Canadian Holstein cattle.

Trait^b^	Sample size	De-regressed breeding values				Reliability	
		Mean^a^	Minimum	Maximum	SD	Mean	SD
FL	22,284	1.82	−13.82	17.64	5.78	0.57	0.19
FAN	20,014	20.41	−121.01	202.26	39.70	0.53	0.19
HDe	23,266	1.10	−20.35	27.87	4.48	0.47	0.16
BCS	22,966	99.69	80.42	113.44	3.73	0.52	0.12
BD	22,471	0.84	−15.09	17.07	4.97	0.55	0.10
DC	23,269	0.16	−18.72	20.09	5.80	0.48	0.08
CW	22,706	1.28	−12.53	15.31	4.28	0.55	0.16
HFE	15,269	2.42	−15.23	18.09	4.65	0.47	0.03
ST	20,387	31.53	−144.46	247.63	54.12	0.65	0.07
BQ	23,512	2.12	−18.38	33.66	4.36	0.55	0.13
FLV	20,890	29.57	−126.81	241.57	48.02	0.47	0.17
LOC	24,893	3.22	−5.08	11.14	3.098	0.38	0.15
RLRV	20,168	20.24	−136.60	234.53	40.22	0.53	0.20
RLSV	20,762	29.02	−126.12	237.32	47.62	0.56	0.14

SD, standard deviation;

^a^ The EBV, for BCS, were scaled to an average of 100 and SD, equal to 5, while all other trait EBVs, were scaled to an average of zero and SD, equal to 5.

^b^ All abbreviations are defined in Table 1.

### 2.3 Genotype imputation and quality control

Genotype imputation was performed from a MD SNP panel containing 44,315 SNPs to a HD SNP panel containing 311,725 SNPs [after a preliminary quality control (Chen et al., 2022)]. The accuracy of genotype imputation for this population was >0.93 (Larmer et al., 2017). In the study, MD genotypes were available for 39,135 animals, comprising 24,721 females and 14,414 males. Meanwhile, the reference population with HD data consisted of 2,507 Holstein animals, which included 562 females and 1,945 males. Before genotype imputation, the SNPs present only in the MD SNP panel were excluded from further analyses. Moreover, a quality control (QC) was performed using the PLINK 1.9 software (Purcell et al., 2007) to remove SNPs: 1) with call rate <0.95; 2) with an extreme deviation from Hardy-Weinberg equilibrium (*p* < 10^–8^) as an indication of genotyping errors; 3) located in non-autosomal chromosomes; and 4) with unknown genomic position based on the ARS-UCD1.2 reference genome assembly. After QC, 40,442 and 2,94,671 SNPs remained in the MD and HD panels, respectively. The genotype phasing was performed using the Eagle 2.4.1 software (Loh et al., 2016), while genotype imputation was done using the Minimac4 software (Das et al., 2016). After genotype imputation, an additional QC was performed to exclude individuals or SNPs with call rate <0.90, SNPs with minor allele frequency (MAF) < 0.01, and SNPs with extreme deviation from Hardy-Weinberg equilibrium (*p* < 10^–8^). Finally, the number of SNPs that remained for the GWAS analyses ranged between 2,84,289 for HDe and 2,87,048 for RLSV.

### 2.4 Genome-wide association analyses

Mixed linear model (MLM) analyses were performed to estimate the SNP effects using the GCTA package (Yang et al., 2011). The univariate model used can be described as follows:

y=μ+Xβ+Za+e,

where y is the vector of dEBVs for each trait; μ is the overall mean; β is the fixed effect of the SNP being tested for association with each trait (i.e., the coefficient of the linear regression on the recoded SNP genotypes: 0, 1 or 2), a is a vector of random polygenic effects $\text{with }a\;∼\;N\left(0,{G\sigma }_{a}^{2}\right),$ where **G** is the genomic-based relationship matrix (**GRM)** (VanRaden, 2008) and $\sigma_{a}^{2}$ is the additive genetic variance; X and **Z** are the incidence matrices for the effects in β and a , respectively; and e is a vector of residuals with $e\;∼\;N\left(0,I\sigma_{e}^{2}\right)$ , where **I** is an identity matrix and $\sigma_{e}^{2}$ is the residual variance.

We used the MLMA-LOCO (mixed linear model association–leave-one-chromosome-out) to avoid the tested marker being adjusted twice in the model as a fixed effect (target SNP) and random effect (in the GRM) (Fraslin et al., 2022). In the MLMA-LOCO method the chromosome in which the SNP being tested is located was excluded when calculating the GRM (Yang et al., 2014). This approach has been increasingly used in livestock studies (e.g., Uzzaman et al., 2018; Sölzer et al., 2022; Kim et al., 2024) to improve the statistical power of the studies. Therefore, 29 GRMs were alternatively constructed by randomly sampling 50,000 SNPs homogeneously distributed across the genome (after QC). The SNPs from the MD SNP panels were not used for the GRM because the HD panel had a more homogeneous distribution of SNPs across the genome. After the GWAS analyses, all SNPs were ranked based on their *p*-values and clumped according to their linkage disequilibrium (LD) level ( $r^{2}\;>\;0.9$ ). The clumping strategy has been suggested as a preferable approach as compared to the traditional LD pruning strategy (Privé et al., 2018). In the latter strategy, SNPs were sorted according to their statistical importance and only the most significant SNP per region of the genome were kept for further analyses. The genomic inflation factor (λ) was calculated as $\lambda =\text{median}\left(\chi 2\right)/0.456$ (Bacanu et al., 2000) and 95% confidence intervals for the λ values were derived.

### 2.5 Correction for multiple tests

As many variants were tested, the traditional Bonferroni correction would be highly conservative as not all tests are independent due to LD among markers (Johnson et al., 2010). Thus, to avoid excessive false-negative results, a modified Bonferroni correction was applied by using the number of independent chromosomal segments (Me) at the genome-wide level (Li et al., 2015) instead of the total number of SNPs. The Me is a function of both effective population size (Ne) and genome length (L) in Morgans and it was calculated as $\text{Me}=\left(2\times \text{Ne}\times L\right)/\log\left(\text{Ne}\times L\right)$ (Goddard et al., 2011). One cM was assumed to be equivalent to one Mbp (Wang et al., 2016) Ne was assumed to be 66, as it was the most conservative value recently reported for the same Holstein cattle population (Makanjuola et al., 2020). A SNP effect was considered to be statistically significant if its *P*-value was smaller than the modified Bonferroni genome-wide threshold, i.e. 0.05/Me. The value of Me and the corresponding–log_10_ of significance threshold used were 2,044.7 and 4.61, respectively. This approach has been used in various other studies in the literature (Ricard et al., 2017; van den Berg et al., 2019; Jin et al., 2023). For completeness, we also performed a Bonferroni correction considering a much stricter threshold, which was the same for all traits (6.68), based on the total number of tests performed (0.05/number of informative SNPs for each trait after LD-based clumping). Some disadvantages of this much stricter Bonferroni threshold are that it does not consider the dependence among the tests due to linkage disequilibrium between the SNPs and it is known for being less tolerant to type I errors than other adaptations of this method or other methods. Therefore, many biologically important associations might not be identified when considering this strict Bonferroni correction (i.e., higher incidence of type II errors–false negatives).

### 2.6 Functional genomic analyses

The SNP coordinates were based on the ARS-UCD1.2 assembly of the cattle reference genome available in the GenBank accession (GCA_002263795.2). The GALLO R package (Fonseca et al., 2020) was used to detect positional genes and quantitative trait loci (QTL) located 100 Kb upstream or downstream of each significant SNPs (threshold defined based on the linkage disequilibrium level in the studied population (Sargolzaei et al., 2008). The QTL database used was the Animal QTLdb Release 49 (Hu et al., 2019). Subsequently, functional enrichment analyses were performed for the genes found for each trait separately, using the DAVID platform (Huang et al., 2009). A False Discovery Rate (FDR) of 0.05 was used in the functional enrichment analyses to control false discoveries over the multiple tests, it is an option for filtering the results provided by the DAVID platform that requests *p*-values adjusted by adaptive linear increment for approximate control of the FDR (Benjamini and Hochberg, 2000).

## 3 Results

### 3.1 Association analyses

After QC and LD-based clumping, the remaining number of informative SNPs (and animals) were 241,552 (22,966) for BCS; 242,222 (22,471) for BD; 240,263 (23,512) for BQ; 241,290 (23,269) for DC; 242,163 (22,706) for CW; 242,419 (22,284) for FL; 240,781 (20,014) for FAN; 239,533 (20,890) for FLV; 240,140 (23,266) for HDe; 242,264 (15,269) for HFE; 241,443 (24,893) for LOC; 240,305 (20,168) for RLRV; 242,747 (20,762) for RLSV, and 242,512 (20,387) for ST. The λ values ranged from 1.01 to 1.05 and the Q-Q plots for all traits are shown in Supplementary File S1 (dx.doi.org/10.6084/m9.figshare.27160302).

Detailed information about the GWAS, including *p*-values, SNP effects, MAF, QTL, and candidate genes found within an interval of 100 Kb upstream and downstream from the significant SNPs are provided in Supplementary File S2 (dx.doi.org/10.6084/m9.figshare.27160302). Table 3 shows a summary of the main significant findings of SNPs and genes. A total of 20, 60, 13, 17, 27, 13, 8, 7, 19, 4, 10, 13, 15, and 7 significant SNPs were identified for BCS, BD, BQ, CW, DC, FL, FAN, FLV, HDe, HFE, LOC, RLRV, RLSV, and ST, respectively. No significant SNPs were found on BTA24, BTA27, and BTA29 for any trait evaluated. On the other hand, BTA20 had the highest number of significant SNPs (n = 64). The BTA9 and BTA11 harbored significant SNPs for the highest number (n = 6) of traits. The SNP rs137570291 (BTA11) was the only SNP associated with more than one trait (BD and LOC). Only markers rs41936372 (BTA20: 11,927,432 bp) for HDe, rs110434046(BTA6: 87,184,768 bp) for BCS and rs108938667 (BTA11: 76,875,084 bp) for BD were significant when considering a stricter threshold for the Bonferroni correction for multiple tests. Table 4 presents the summary of the main positional genes associated with significant markers. Supplementary Figures S1–S6 present the Manhattan plots for all traits and the Supplementary Tables S1–S9, with significative SNPs and genes, are shown in Supplementary File S3 (dx.doi.org/10.6084/m9.figshare.27160302).

**TABLE 3:** Summary of the most significant Single Nucleotide Polymorphisms (SNPs) associated with each body conformation trait in Canadian Holstein cattle. The full list is presented in the Supplementary File S2.

Trait	Chr	Variant	Location (bp)	MAF	Effect	*P*-value	Genes
FL	BTA13	rs109648982*	50,448,257	0.418	0.439	1.514 × 10^−05^	*ENSBTAG00000007199*
FAN	BTA12	rs133014265*	12,322,089	0.063	−7.737	1.310 × 10^−06^	*ENSBTAG00000053271, DGKH, AKAP11*
HDe	BTA20	rs41936372**	11,927,432	0.469	−0.714	8.917 × 10^−08^	*—*
BCS	BTA6	rs110434046**	87,184,768	0.410	−0.987	4.262 × 10^−08^	*NPFFR2*
BD	BTA11	rs108938667**	76,875,084	0.486	−0.789	5.640 × 10^−08^	*—*
DC	BTA8	rs43571286*	78,118,013	0.137	1.140	3.566 × 10^−07^	*NTRK2*
CW	BTA28	rs42139508*	23,422,787	0.375	0.586	4.802 × 10^−07^	*CTNNA3, LRRTM3*
HFE	BTA17	rs137254844*	42,276,117	0.025	−4.772	5.667 × 10^−06^	*PDGFC*
ST	BTA1	BOVINEHD0100039562*	156,730,566	0.048	10.368	7.770 × 10^−07^	*KCNH8*
BQ	BTA19	rs136174626*	27,056,493	0.219	−0.925	2.530 × 10^−06^	*CTDNEP1, ELP5, CLDN7, SLC2A4, YBX2, EIF5A, GPS2, NEURL4, ENSBTAG00000045892, KCTD11, TMEM95, TNK1, PLSCR3, TMEM256, NLGN2, SPEM1, SPEM2, TMEM102, ENSBTAG00000050569, CHRNB1, ZBTB4, POLR2A*
FLV	BTA11	rs136468307*	39,657,165	0.079	−12.325	9.448 × 10^−07^	*—*
RLRV	BTA15	rs41781092*	75,567,845	0.285	−6.427	1.767 × 10^−07^	*ENSBTAG00000054083*
RLSV	BTA7	rs3423241779*	28,761,924	0.171	4.058	3.132 × 10^−06^	*—*

BTA, *Bos taurus* autosome; Chr, chromosome; MAF, minor allele frequency; *Significant SNPs, based only on the Bonferroni correction considering the number of independent chromosomal segments; **Significant SNPs, based on a stricter threshold for Bonferroni multiple testing correction.

**TABLE 4:** Summary of the main positional genes associated with significant markers.

Chr	Genes	Associated trait	References
Composite feet and legs score index			
BTA13	*DIP2C*	Claw lesions	Lai et al. (2021)
	*DIP2C*	Lameness and conformation	Ring et al. (2018)
	*BIRC7*	Osteogenic differentiation	Liu et al. (2017)
	*COL20A1*	Collagen formation	Rajasekaran et al. (2023)
	*YTHDF1*	Osteoporosis	Liu et al. (2021)
BTA17	*MN1*	Craniofacial development	Breckpot et al. (2016); Hoebel et al. (2017)
BTA18	*FGF21*	Bone mass	Wei et al. (2012)
	*PPP1R15A*	Bone development	Ding et al. (2024)
	*BAX*	Bone development	Zaman et al. (2012)
	*EMC10*	Body mass index	Wang et al. (2022)
Foot Score Traits			
BTA11	*RSAD2*	Viral replication	Yogarajah et al. (2018)
BTA12	*DGKH*	Regulating the growth	Lu et al. (2020)
BTA15	*LRP4*	Congenital syndactyly	Drögemüller et al. (2007)
Mobility Score Traits			
BTA1	*RYK*	Shortened long bones	Andre et al. (2012)
BTA4	*EXOC4*	Meat quality	Bordbar et al. (2019)
	*EXOC4*	Temperament traits	Ruiz-De-La-Cruz et al. (2023)
BTA5	*FGF23*	Subclinical hypocalcemia	Simic and Babitt (2021)
	*FGF23*	1,25-Dihydroxyvitamin D synthesis	Ma et al. (2022)
BTA14	*STMN2*	Muscle atrophy	Guerra San Juan et al. (2022)
BTA19	*MAP2K6*	Adaptive thermogenesis	Ryu et al. (2012)
Dairy Strength Score Traits			
BTA3	*ALDH9A1*	Carnitine synthesis	Schlegel et al. (2012)
	*ALDH9A1*	Fat metabolism	Li et al. (2022)
	*PDE4B*	Milk yield	Lee et al. (2015)
	*PDE4B*	Protein content	Kim et al. (2021)
BTA5	*ABCC9*	Udder depth and fore udder attachment	Tribout et al. (2020)
	*ABCC9*	Fertility	Nayeri et al. (2016)
	*ABCC9*	Milk fatty acids	Jiang et al. (2019)
	*LGALS1*	Maternal-conceptus immune tolerance	Chaney et al. (2022)
BTA6	*GC*	Clinical mastitis resistance	Lee et al. (2021)
	*CSN1S1*	Protein content	Korkuć et al. (2023)
BTA11	*MRPS5*	First *postpartum* anoestrus interval	Melo et al. (2019)
BTA20	*GHR*	Calf birth weigh	Hartati et al. (2019)
	*GHR*	Milk fatty acids	Jiang et al. (2019)
	*GHR*	Fat yield	Li et al. (2014)
	*OXCT1*	Fat yield	Li et al. (2014)
	*RPL37*	Protein content	Oliveira et al. (2019)

### 3.2 Composite feet and legs score index

Thirteen SNPs distributed across the chromosomes BTA11, BTA13, BTA17, BTA18, and BTA26 (Supplementary Tables S1, in Supplementary File S3; (dx.doi.org/10.6084/m9.figshare.27160302) were significantly associated with FL. The most statistically significant SNP (rs41661000) for FL is located on BTA13: 50,453,781 bp. Fifty-two candidate genes with known biological functions (Supplementary Tables S1 in Supplementary File S3; dx.doi.org/10.6084/m9.figshare.27160302) were found near the significant SNPs, but no genes were identified close to the significant SNPs on BTA26. Moreover, various QTL for FL, FAN, RLSV, ST, abomasum displacement, body weight, feed conversion ratio, residual feed intake, and udder depth were previously reported in the same genomic regions of the SNPs associated with FL. Three significant gene ontology (GO) terms were associated with FL (Supplementary Tables S2 in Supplementary File S3; dx.doi.org/10.6084/m9.figshare.27160302), and three genes associated with FL are involved with these GO terms.

### 3.3 Foot score traits

Twenty-seven SNPs were found to be associated with foot score composition traits (Supplementary File S3; dx.doi.org/10.6084/m9.figshare.27160302). These SNPs are located on BTA1, BTA2, BTA4, BTA7, BTA9, BTA10, BTA11, BTA12, BTA15, BTA19, and BTA20. The most statistically significant SNPs for FAN and HDe were rs133014265 $\left(\text{BTA}12:12,322,089\text{ bp}\right)$ and rs41936372 $\left(\text{BTA}20:11,927,432\text{ bp}\right)$ , respectively. The genomic regions around these SNPs harbor 18 candidate genes for FAN and 22 for HDe (Supplementary Tables S3 in Supplementary File S3; dx.doi.org/10.6084/m9.figshare.27160302). Several QTL were previously reported in the same genomic regions of the SNPs, including QTL for ANG, BD, FAN, HDe, FL, RLSV, ST, body height, body weight, bone percentage, carcass weight, conformation score, dairy form, immunoglobulin g level, teat length, and teat placement (Supplementary File S2; dx.doi.org/10.6084/m9.figshare.27160302). Fourteen significant GO terms were identified for FAN, but no significant GO terms were found for HDe. Supplementary Tables S4 (Supplementary File S3; dx.doi.org/10.6084/m9.figshare. 27160302) presents the 11 genes enriched for 14 GO terms for FAN, which contains KEGG pathways, such as protein families, molecular functions, biological processes, and cellular components related to olfactory receptor, sensory transduction, and cell membrane.

### 3.4 Mobility score traits

Fifty-eight SNPs located across 19 chromosomes were found to be significantly associated with mobility composition score traits (Supplementary File S2; dx.doi.org/10.6084/m9.figshare.27160302). The most statistically significant SNPs for BQ, FLV, LOC, RLRV, and RLSV were: rs136174626 $\left(\text{BTA}19:27,056,493\text{ bp}\right)$ , rs136468307 $\left(\text{BTA}11:39,657,165\text{ bp}\right)$ , rs137570291 $\left(\text{BTA}11:76,829,758\text{ bp}\right)$ , rs41781092 $\left(\text{BTA}15:75,567,845\text{ bp}\right)$ , and rs3423241779 $\left(\text{BTA}7:28,761,924\text{ bp}\right)$ . These genomic regions harbor 45, 4, 9, 16, and 16 candidate genes associated with BQ, FLV, LOC, RLRV, and RLSV, respectively (Supplementary Tables S5, S6 in Supplementary File S3; dx.doi.org/10.6084/m9.figshare.27160302). Moreover, these regions harbor QTL previously associated with various traits, such as ANG, BD, BQ, FL, FAN, HDe, RLRV, RLSV, ST, body weight, chest depth, conformation score, dairy form, immunoglobulin g level, and teat length (Supplementary File S2; dx.doi.org/10.6084/m9.figshare.27160302). Five significant GO terms were associated with composite mobility score traits, including one for BQ and four for RLSV. No GO terms were enriched for FLV, LOC, and RLRV. Supplementary Table S2 (Supplementary File S3; dx.doi.org/10.6084/m9.figshare.27160302), describes the 14 genes that were enriched for the GO term for BQ. Whereas seven genes were enriched for the four GO terms for RLSV (Supplementary Table S2 in Supplementary File S3; dx.doi.org/10.6084/m9.figshare.27160302).

### 3.5 Dairy strength score traits

A total of 135 SNPs located on 21 chromosomes were found to be associated with dairy strength traits (Supplementary File S2; dx.doi.org/10.6084/m9.figshare.27160302). The most statistically significant SNPs for BCS, BD, CW, DC, HFE, and ST were rs110434046 $\left(\text{BTA}6:87,184,768\text{ bp}\right)$ , rs108938667 $\left(\text{BTA}11:76,875,084\text{ bp}\right)$ , rs42139508 $\left(\text{BTA}28:23,422,787\text{ bp}\right)$ , rs43571286 $\left(\text{BTA}8:78,118,013\text{ bp}\right)$ , rs137254844 $\left(\text{BTA}17:42,276,117\text{ bp}\right)$ , and BOVINEHD0100039562 $\left(\text{BTA}1:156,730,566\text{ bp}\right)$ , respectively. The genomic regions around the significant SNPs harbor 24 candidate genes for BCS, 37 for BD, 27 for CW, 31 for DC, 9 for HFE, and 20 for ST (Supplementary Tables S7–S9 in Supplementary File S3; dx.doi.org/10.6084/m9.figshare.27160302). Moreover, several QTL were previously reported in the same genomic regions, including QTL for BCS, BD, CW, DC, FAN, HDe, RLRV, RLSV, ST, average daily gain, body height, body size, body weight, bone percentage, calf size, dairy form, dry matter intake, fertility index, immunoglobulin G level, interdigital hyperplasia, lactation persistency, and sole ulcer. Five significant GO containing three genes in common were identified for BCS (Supplementary Table S2 in Supplementary File S3; dx.doi.org/10.6084/m9.figshare.27160302), but no significant terms were found for the other dairy strength traits.

## 4 Discussion

### 4.1 Composite feet and legs score index

The composite Feet and Leg score index is a weighted linear combination of foot and leg conformation traits used as a selection sub-index for genetically improving these traits in Canadian Holstein cattle. Fifty-two positional candidate genes were harboring or near the 13 SNPs associated with this index. In the significant regions, BTA13 harbors the genes *DIP2C* (disco-interacting protein 2 homolog C), *BIRC7* (Baculoviral IAP repeat-containing 7), *COL20A1* (Collagen Type XX Alpha 1 Chain), and *YTHDF1* (YTH N6-Methyladenosine RNA Binding Protein F1). The *DIP2C* gene has been reported to be associated with sole ulcers, white line disease, and non-infectious claw lesions in Holstein cattle (Lai et al., 2021). Ring et al. (2018) reported that hoof diseases were genetically correlated to lameness and conformation in Irish cows. Moreover, the *BIRC7* gene is associated with the osteogenic differentiation of human cells (Liu et al., 2017), while *COL20A1* is associated with human collagen formation (Rajasekaran et al., 2023). Liu et al. (2021) reported that *YTHDF1* is related to osteoporosis, and its expression increases human bone marrow mesenchymal stem cells during osteogenic differentiation. Furthermore, *YTHDF1* knockout mice result in decreased bone mass *in vivo*. Factors that affect bone density, such as diseases or their differentiation processes, may be related to FL in cows, as an association has already been found between bone ratio and visual conformation assessments (Kempster et al., 1982) in different dairy cattle breeds.

The *MN1* (MN1 Proto-Oncogene, Transcriptional Regulator) gene, located on BTA17, has been linked to craniofacial development in mice and humans (Breckpot et al., 2016; Hoebel et al., 2017). On BTA18, the genes *FGF21* (Fibroblast Growth Factor 21), *PPP1R15A* (Protein Phosphatase 1 Regulatory Subunit 15A), *BAX* (BCL2 Associated X, Apoptosis Regulator), and *EMC10* (ER Membrane Protein Complex Subunit 10) were identified as associated with FL. Wei et al. (2012) identified *FGF21* as a physiologically and pharmacologically significant negative regulator of bone mass. The *PPP1R15A* and *BAX* genes were also previously associated with bone development in mice and humans (Zaman et al., 2012; Ding et al., 2024), while Wang et al. (2022) reported that *EMC10* serum levels are correlated to body mass index (BMI) and insulin resistance in humans and as a potential biomarker of adiposity, in mice and humans. Onyiro and Brotherstone (2008) reported an association of bone mass with leg problems in Holstein-Friesian dairy cows. Therefore, bone mass can potentially affect FL and other conformation traits.

Various QTL (Supplementary File S2; dx.doi.org/10.6084/m9.figshare.27160302) were previously reported to be located on the genomic regions around the SNPs significantly associated with FL. Cole et al. (2011) identified the SNPs rs134739530 (BTA3:55,365,713 bp) and rs134119868 (BTA3:55,513,275 bp) associated with QTL for FL, FAN, RLSV, ST, and udder depth in the same regions where SNP associated with FL in Holstein cows in the current study. Moreover, some production QTL were found in these genomic regions (body weight, feed conversion ratio, residual feed intake). Pérez-Cabal and Charfeddine (2016) indicated that high body weight was linked to a higher probability of disorders in the conformation of feet and legs in Spanish Holstein cows.

### 4.2 Foot score traits

Two foot score traits (FAN and HDe) were included in this study, and 40 positional candidate genes (Supplementary Table S3 in Supplementary File S3; dx.doi.org/10.6084/m9.figshare.27160302) were found to be associated with these traits. Yogarajah et al. (2018) reported that increased expression of the *RSAD2* (Radical S-Adenosyl Methionine Domain Containing 2) gene, located on BTA11, was correlated to reduced viral replication of Coxsackievirus A16 (CV-A16). This enterovirus causes diseases in the human hand, foot, and mouth. The *DGKH* (Diacylglycerol Kinase Eta) gene*,* located on BTA12, was reported as a candidate gene expressed in the pituitary gland for regulating the growth of Yunling and Leiqiong cattle through the secretion of growth-related hormones (Lu et al., 2020). The *LRP4* (LDL Receptor Related Protein 4) gene, located on BTA15, has been associated with congenital syndactyly in Holstein cattle (Drögemüller et al., 2007). This is an autosomal recessive abnormality characterized by the fusion of the functional digits (Duchesne et al., 2006).

QTL for bone percentage and carcass weight, milk yield, body weight, and conception rate were previously reported (Supplementary File S2; dx.doi.org/10.6084/m9.figshare.27160302) to overlap with the genomic region identified for foot score traits. These QTL may be influenced by injuries to the foot regions that lead to reduced milk yield, lack of weight gain, poor fertility, and, consequently, animal culling rates (Garvey, 2022). Cole et al. (2011) identified a SNP (rs41628909, BTA1:67,619,208 bp) associated with a QTL for FAN, which was identified to be associated with HDe in the present study. Boichard et al. (2003) identified a SNP (rs137103653, BTA7:28,218,213 bp) associated with HDe, which in this study was identified for FAN. According to Pérez-Cabal and Charfeddine (2016), a high score for FAN means that cows have a steep angle and require less frequent hoof trimming. However, the extremely steep foot angle can interfere with the adequate cushioning effect on the coronary band and can place undue stress on the junction between the wall and the sole of the claw. Furthermore, HDe is the depth of the heel on the outside claw and when its measurement is extremely shallow it was associated with a much higher than average incidence of horn lesions (Chapinal et al., 2013).

Eleven GO terms identified are related to olfactory transducers or receptors. Olfaction is an ancient sensory system that allows organisms to detect chemicals in their environment, and the first step in odor transduction is mediated by binding odorants to olfactory receptors (Gaillard et al., 2004). According to Padodara and Jacob (2014), cattle use the sense of smell to complement their visual information, and social group organization to recognize individual animals and create bonds between mother and offspring. The olfactory communication between animals and reproduction is based mainly on released pheromones, in addition to the fact that in the food search, the odor can condition the animal’s appetite (Padodara and Jacob, 2014).

### 4.3 Mobility score traits

Ninety positional candidate genes were identified for the five mobility score traits (BQ, FLV, LOC, RLRV, and RLSV) included in this study (Supplementary Tables S9, S10 in Supplementary File S3; dx.doi.org/10.6084/m9.figshare.27160302). Noteworthy genes include RYK (Receiver Like Tyrosine Kinase), EXOC4 (Exocyst Complex Component 4), FGF23 (Fibroblast Growth Factor 23), STMN2 (Stathmin 2), and MAP2K6 (Mitogen-Activated Protein Kinase Kinase 6), located on BTA1, BTA4, BTA5, BTA14, and BTA19, respectively. The RYK gene was previously associated with shortened long bones in the limbs in mice (Andre et al., 2012), while FGF23 was associated with subclinical hypocalcemia in dairy cows and 1,25-Dihydroxyvitamin D synthesis (Simic and Babitt, 2021; Ma et al., 2022). The STMN2 gene was associated with muscle atrophy and impaired motor behavior in mouse (Guerra San Juan et al., 2022) and it is a candidate gene for classical Bovine Spongiform Encephalopathy (Thomson et al., 2012). The EXOC4 gene was identified as a candidate gene for meat quality in Simmental cattle (Bordbar et al., 2019) and for temperament traits in Brahman cattle (Ruiz-De-La-Cruz et al., 2023). The MAP2K6 gene was associated with adaptive thermogenesis in cattle (Ryu et al., 2012). These associations may indicate factors that affect cattle mobility because they are related to muscle, bone, neural, and behavioral development.

Nine QTL for conformation traits (ANG, BD, BQ, FL, FAN, HDe, RLRV, RLSV, and ST) were previously identified in the same region found in the present study for mobility score traits. Cole et al. (2011) identified a SNP (rs132818385, BTA20:18,725,426 bp) associated with QTL regions for RLRV, FL, FAN, and ST, which, in the present study, was identified for BQ, a second SNP (rs109011936, BTA11:78,444,403 bp) associated with RLRV, which was associated with LOC in the current study, and a third SNP (rs41627857, BTA12:8,747,286 bp) associated with RLRV, which was also associated with RLRV in the present study. BQ is assessed by the flatness and cleanness of bone in the shank, hock, and thigh regions (Onyiro and Brotherstone, 2008). According to Atkins and Shannon (2002), a high score of BQ reflects bone that is extremely flat with cleanness throughout and tendons well defined. A good score for this trait may indicate that the animal does not present excessive swelling in the joints, having a good fitness and good circulation in the legs. RLSV assesses the degree of curvature of the hock when viewed from the side. Extremely curved legs were associated with a higher-than-average incidence of horn lesions (Chapinal et al., 2013). The RLRV trait evaluates the straightness of the rear legs when viewed from behind and is measured by the degree of inward deviation of the hocks and the corresponding degree to which the toes point outward (Atkins and Shannon, 2002). Sole ulcer is a hoof problem that can potentially reduce mobility in cattle. This is generally the second leading cause of reported hoof-related lameness in Canadian Holstein cattle (Malchiodi et al., 2020) and is one of the most persistent and costly hoof lesion type (Whay et al., 1998; Cha et al., 2010).

### 4.4 Dairy strength score traits

This study included six composite dairy strength traits (BCS, BD, CW, DC, HFE, and ST) and identified 128 positional candidate genes (Supplementary Tables S11–S13 in Supplementary File S3; dx.doi.org/10.6084/m9.figshare.27160302). The ALDH9A1 (Aldehyde Dehydrogenase 9 Family Member A1) and PDE4B (Phosphodiesterase 4B) genes on BTA3, are related to fat metabolism. The ALDH9A1 gene is involved in carnitine synthesis and carnitine uptake in the liver of dairy cows in the transition period and at different stages of lactation (Schlegel et al., 2012), and fat metabolism in bovine fetal fibroblasts (Li et al., 2022). In addition to being involved with fat yield, PDE4B is associated with milk yield and protein content in Holstein cows (Lee et al., 2015; Kim et al., 2021). Accordingly, Oliveira Junior et al. (2021) found a positive correlation between DC and milk yield (0.54 ±0.01), DC and protein yield (0.52 ± 0.01), and DC and fat yield (0.45 ± 0.01).

Other positional candidate genes were located on BTA5 (*ABCC9* - ATP Binding Cassette Subfamily C Member 9; *LGALS1* - Galectin 1), BTA6 (*GC* - GC Vitamin D Binding Protein; *CSN1S1* - Casein Alpha S1), BTA11 (*MRPS5* - Mitochondrial Ribosomal Protein S5) and in BTA20 (*GHR* - Growth Hormone Receptor; *OXCT1* - 3-Oxoacid CoA-Transferase 1; RPL37 - Ribosomal Protein L37). Some of these genes were previously associated with milk yield and fertility traits. *ABCC9* was associated with udder depth and fore udder attachment (Tribout et al., 2020) and GHR with calf birth weight (Hartati et al., 2019). The *ABCC9* gene was also associated with fertility of dairy cows (Nayeri et al., 2016). MRPS5 is a candidate gene for the first *postpartum* anoestrus interval in Nellore and Brahman cattle (Melo et al., 2019). *ABCC9*, *GHR*, and *OXCT1* were associated with fat yield and synthesis of milk fatty acids in Holstein cattle (Li et al., 2014; Jiang et al., 2019) *CSN1S1* and *RPL37* were associated with protein content in German Black Pied (Korkuć et al., 2023) and Ayrshire and Jersey cattle (Oliveira et al., 2019). The *LGALS1* and *GC* genes were previously associated with confer mechanisms of maternal-conceptus immune tolerance (Chaney et al., 2022) and clinical mastitis resistance (Lee et al., 2021) in dairy cattle. In addition to milk yield, already mentioned above, Oliveira Junior et al. (2021) also found correlation with fertility traits, being positive between DC and days open (0.48 ± 0.01) and a negative correlation between BCS and calving to first service (−0.39 ± 0.01).

Previous QTL for conformation traits were reported in the same region where SNPs associated with dairy strength score traits were found. Lee et al. (2021) identified a SNP (rs110310151, BTA6:86,996,470 bp) that was associated with a QTL region for ECC, as also identified in the present study (Supplementary File S2; dx.doi.org/10.6084/m9.figshare.27160302). BCS is a tool to assess dairy cows’ fat reserves, an important factor in dairy cattle management (Roche et al., 2009; Martins et al., 2020). BD was associated with a profitability index (Alcantara et al., 2022), longevity (Zavadilová and Štípková, 2012), feed efficiency (Manafiazar et al., 2016) and fertility (Jagusiak et al., 2014). Ashwell et al. (2005) also identified a SNP (rs29013890, BTA20:34,800,041) associated with a QTL region for BD (Supplementary File S2; dx.doi.org/10.6084/m9.figshare.27160302). An et al. (2020) identified a SNP (rs42848657, BTA11:24,408,850) associated with chest girth, which was associated with CW in the present study. Chest width is measured from the width of chest floor (Canada, 2021). Ashwell et al. (2005) identified a SNP (rs137532092, BTA20:27,329,790 bp) associated with a QTL region for the composite milk capacity index, while in this study it was associated with DC (Supplementary File S2; dx.doi.org/10.6084/m9.figshare.27160302). The classification of DC is done by evaluating the angle of the ribs (direction of the ribs). It is preferable cows with a high spring, angle, and openness of ribs (Canada, 2021). Oliveira Junior et al. (2021) found a negative genetic correlation between ST and age at first service (−0.45 ± 0.01) and a positive genetic correlation with calf size (0.52 ± 0.01). Cole et al. (2011) identified a SNP (rs43345563, BTA3:77,599,781 bp) associated with BD and ST, which in this study was associated with HFE (Supplementary File S2; dx.doi.org/10.6084/m9.figshare.27160302). Significant SNPs associated with ST were also identified in the current study, which overlapped with QTL reported in the literature for the composite milking capacity index (rs110111160, BTA21:60,066,050 bp) (Kolbehdari et al., 2008), PC (BOVINEHD0100039562, BTA1:156730566 bp) (McClure et al., 2010), and chest depth (rs136729009, BTA5:109,344,409 bp) (Boichard et al., 2003).

### 4.5 Limitations, implications, and next steps

This study identified various SNPs, genomic regions, and positional candidate genes associated with conformation traits in Canadian Holstein cattle. The identified SNPs that are not in the MD panels (Supplementary File S4; dx.doi.org/10.6084/m9.figshare.27160302) could be added to MD panels to potentially increase the accuracy of genomic prediction for the traits evaluated. In this study, we first performed multiple testing correction based on the number of independent chromosomal segments, which depends on the effective population size and genome length in Morgans (Goddard et al., 2011). This strategy has been used in various studies in the literature (Ricard et al., 2017; van den Berg et al., 2019; Jin et al., 2023). However, for completeness, we also performed a much stricter Bonferroni multiple testing correction based on the total number of genome-wide markers for each trait (dependent on QC and LD-based clumping and ranged from 239,533 to 242,747 SNPs). This approach considers that all the tests performed are independent, which is not the case as SNPs are in linkage disequilibrium. The Bonferroni method is also well known for being less tolerant with type I errors (false positive associations), and therefore, it could reduce the ability to identify biologically important genomic regions (false negatives). Therefore, we have focused on the most significant SNPs, but also presented the other suggestive SNPs as Supplementary Material. Additional GWAS analyses based on imputed whole-genome sequence data will be performed subsequently as it could enable the identification of additional associations, as has already been done in previous works (Pedrosa et al., 2021; Chen et al., 2022). Furthermore, as there are more SNPs located on the X chromosome when using whole-genome sequence data, adding these SNPs to the analyses would be another important next step, especially in light of recently-published studies (Sanchez et al., 2023). Application of other omics approaches, such as transcriptomics, is recommended to validate the role of identified SNPs and candidate genes.

## 5 Conclusion

The genome-wide association analyses performed in this study enabled the identification of numerous SNPs, located across most of the chromosomes, which are significantly associated with body conformation traits in Holstein cattle. The genomic regions around the significant SNPs overlapped with previously reported QTL for classes of exterior, health, meat and carcass, milk production, and reproduction traits. The candidate genes identified are involved with biological pathways associated with bone development, metabolism, diseases, reproduction, and milk production. These results illustrate the genetic complexity of conformation traits in dairy cattle and contribute to the understanding of the molecular mechanisms underlying the phenotypic expression of body conformation traits in Holstein cattle.

## Data Availability

The original contributions presented in the study are included in the article/Supplementary Material, further inquiries can be directed to the corresponding author.
