# Supplementary material for: Genome-wide association and functional genomic analyses for body conformation traits in North American Holstein cattle
Source: Front Genet. 2024 Oct 24;15:1478788. doi: 10.3389/fgene.2024.1478788 (PMC11540798; doi:10.3389/fgene.2024.1478788)
Supplement: Supplementary file 2 [file Table1.docx]

**SUPPLEMENTARY MATERIAL**


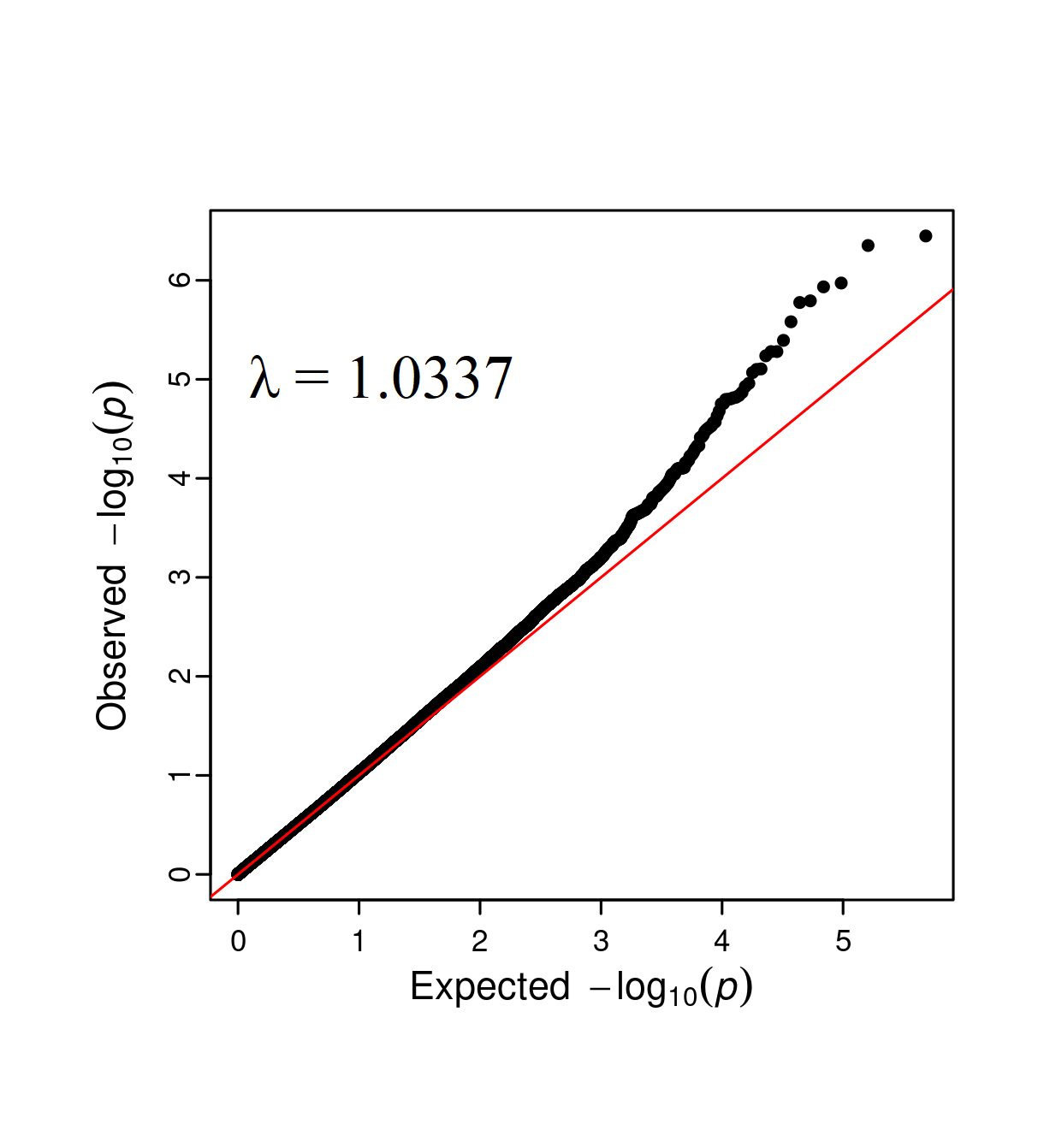


Figure S1. Q-Q plot and λ value of the genome-wide association analysis results for dairy capacity using high-density single nucleotide polymorphism genotypes in Canadian Holstein cattle.


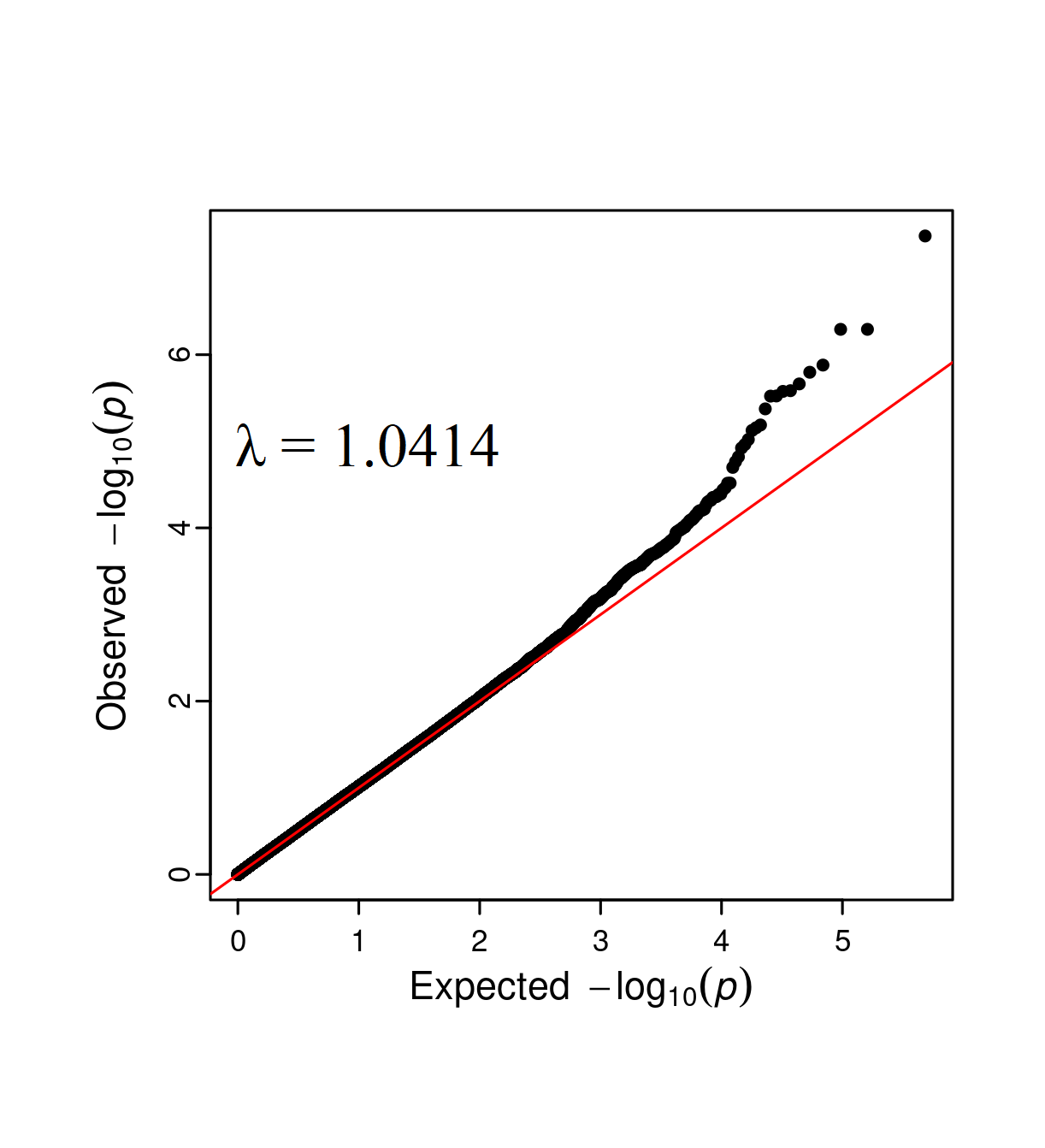


Figure S2. Q-Q plot and λ value of the genome-wide association analysis results for body condition score using high-density single nucleotide polymorphism genotypes in Canadian Holstein cattle.


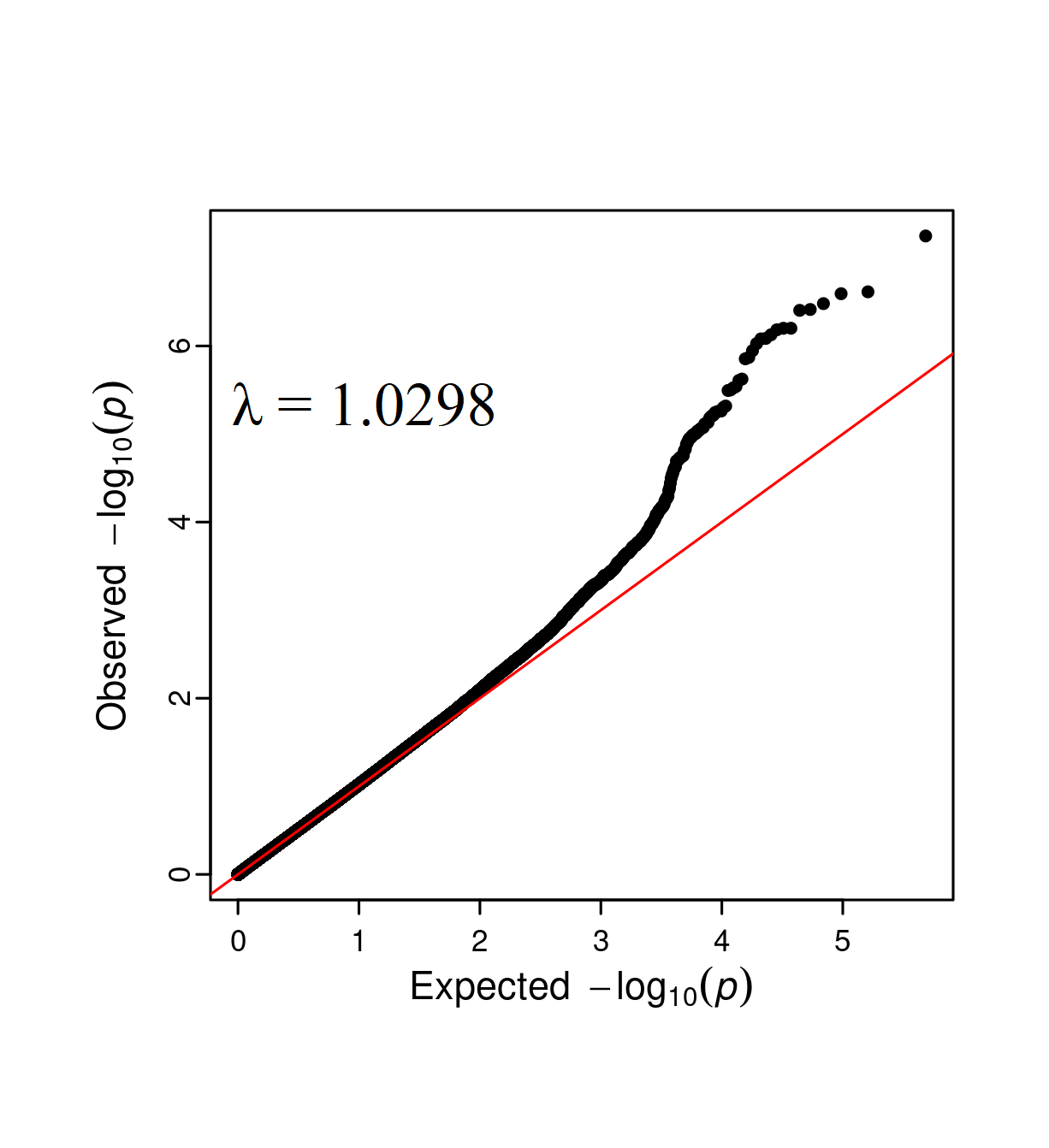


Figure S3. Q-Q plot and λ value of the genome-wide association analysis results for body depth using high-density single nucleotide polymorphism genotypes in Canadian Holstein cattle.


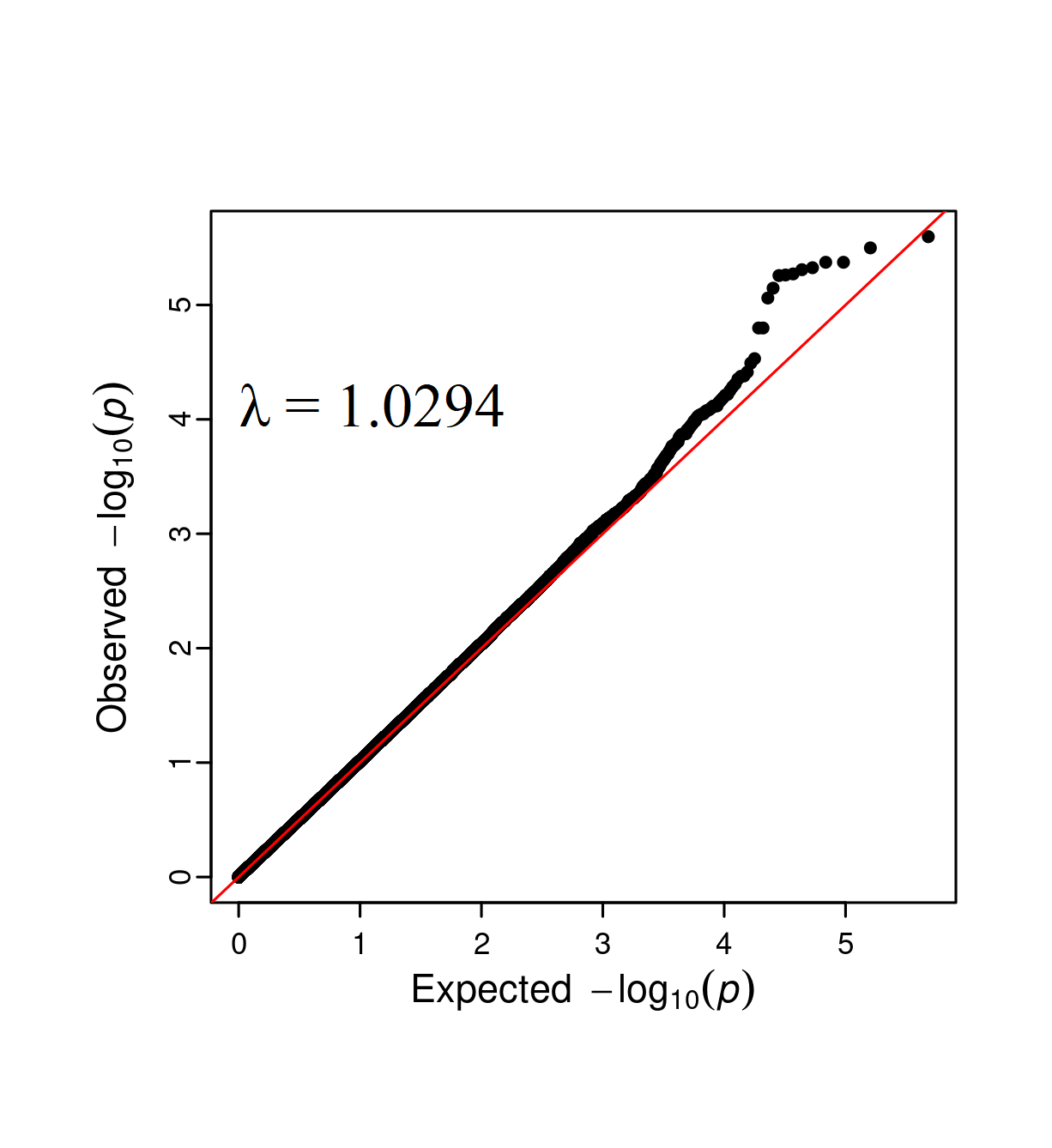


Figure S4. Q-Q plot and λ value of the genome-wide association analysis results for bone quality using high-density single nucleotide polymorphism genotypes in Canadian Holstein cattle.


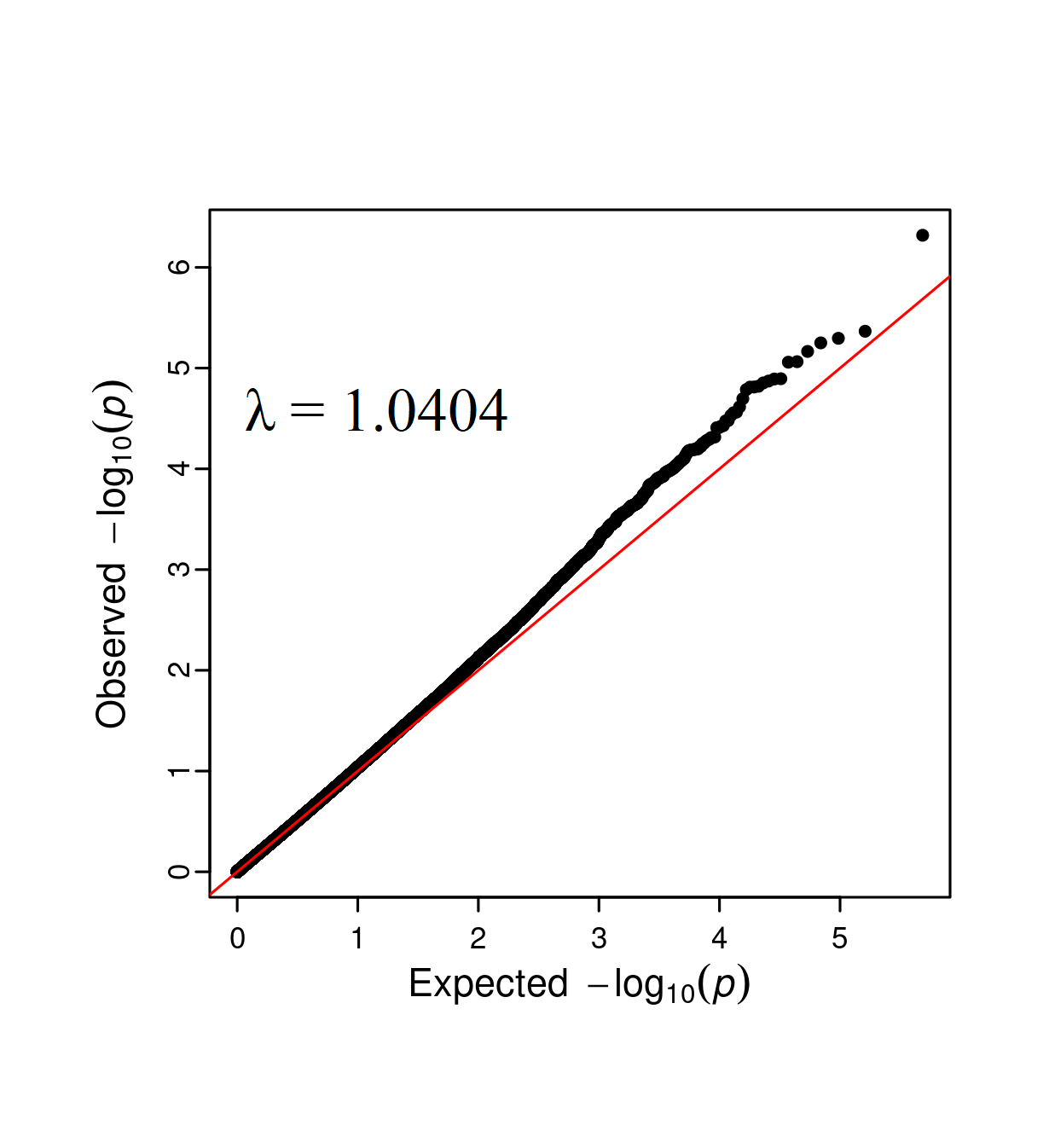


Figure S5. Q-Q plot and λ value of the genome-wide association analysis results for chest width using high-density single nucleotide polymorphism genotypes in Canadian Holstein cattle.


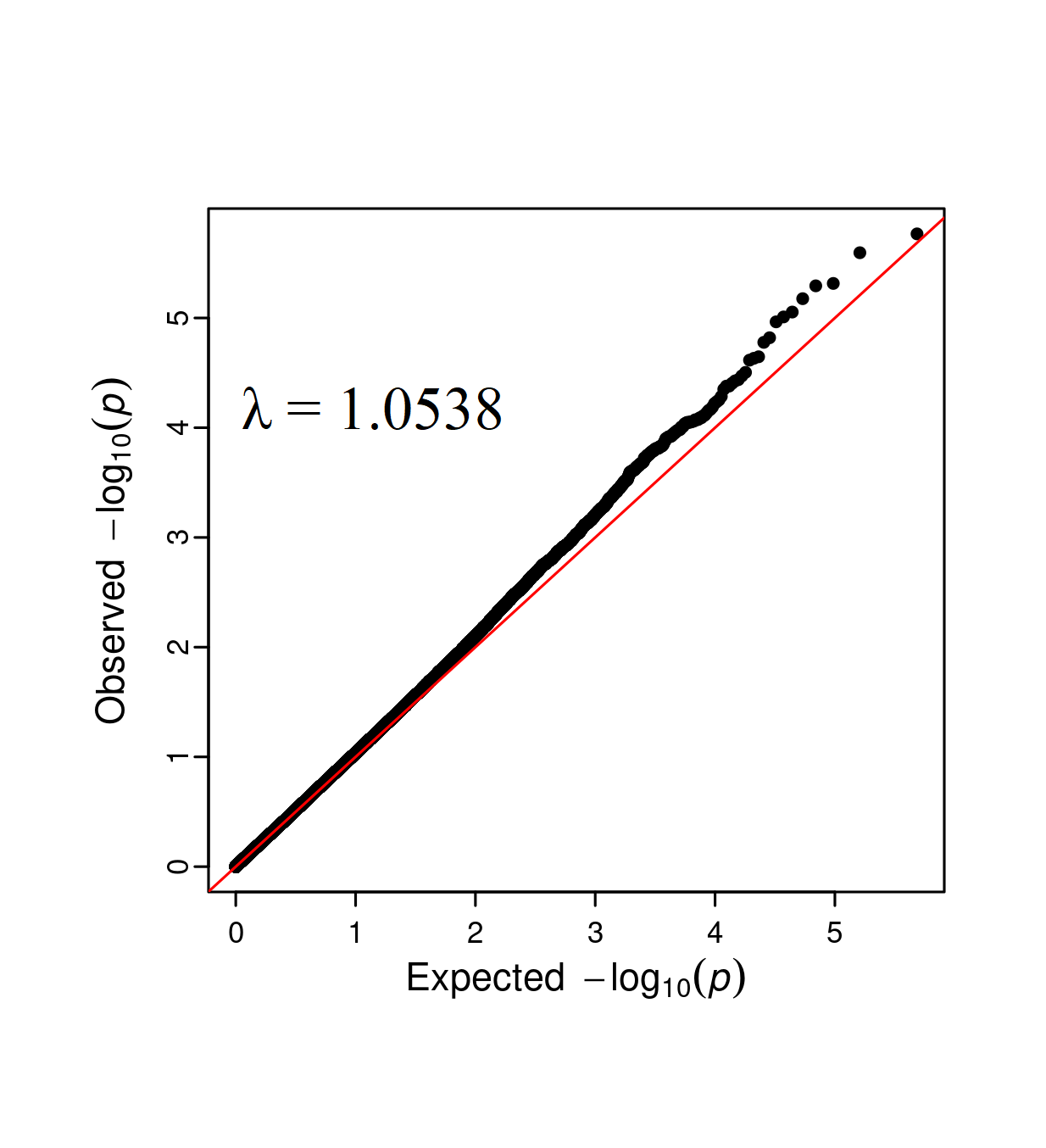


Figure S6. Q-Q plot and λ value of the genome-wide association analysis results for feet and legs using high-density single nucleotide polymorphism genotypes in Canadian Holstein cattle.


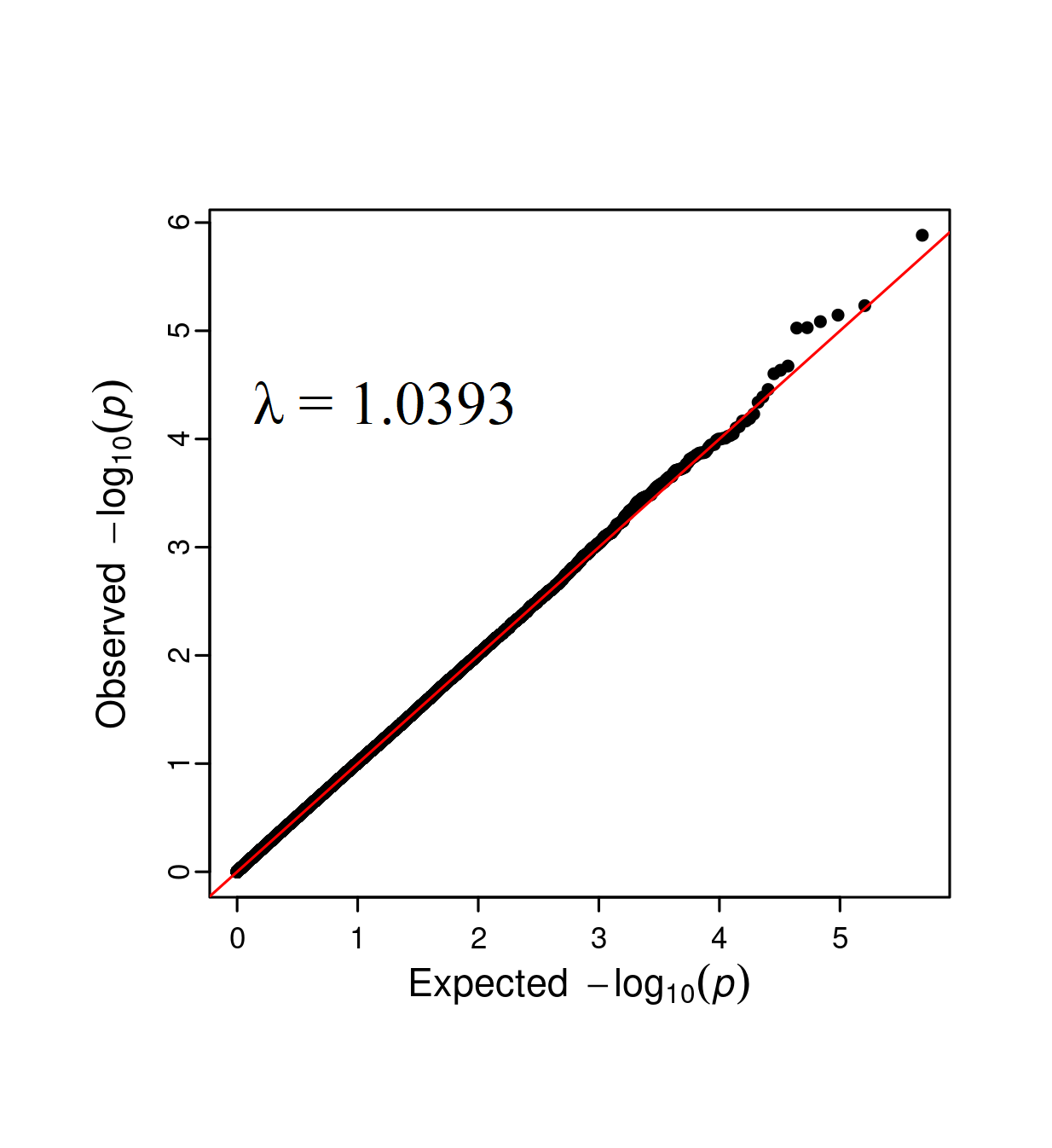


Figure S7. Q-Q plot and λ value of the genome-wide association analysis results for foot angle using high-density single nucleotide polymorphism genotypes in Canadian Holstein cattle.


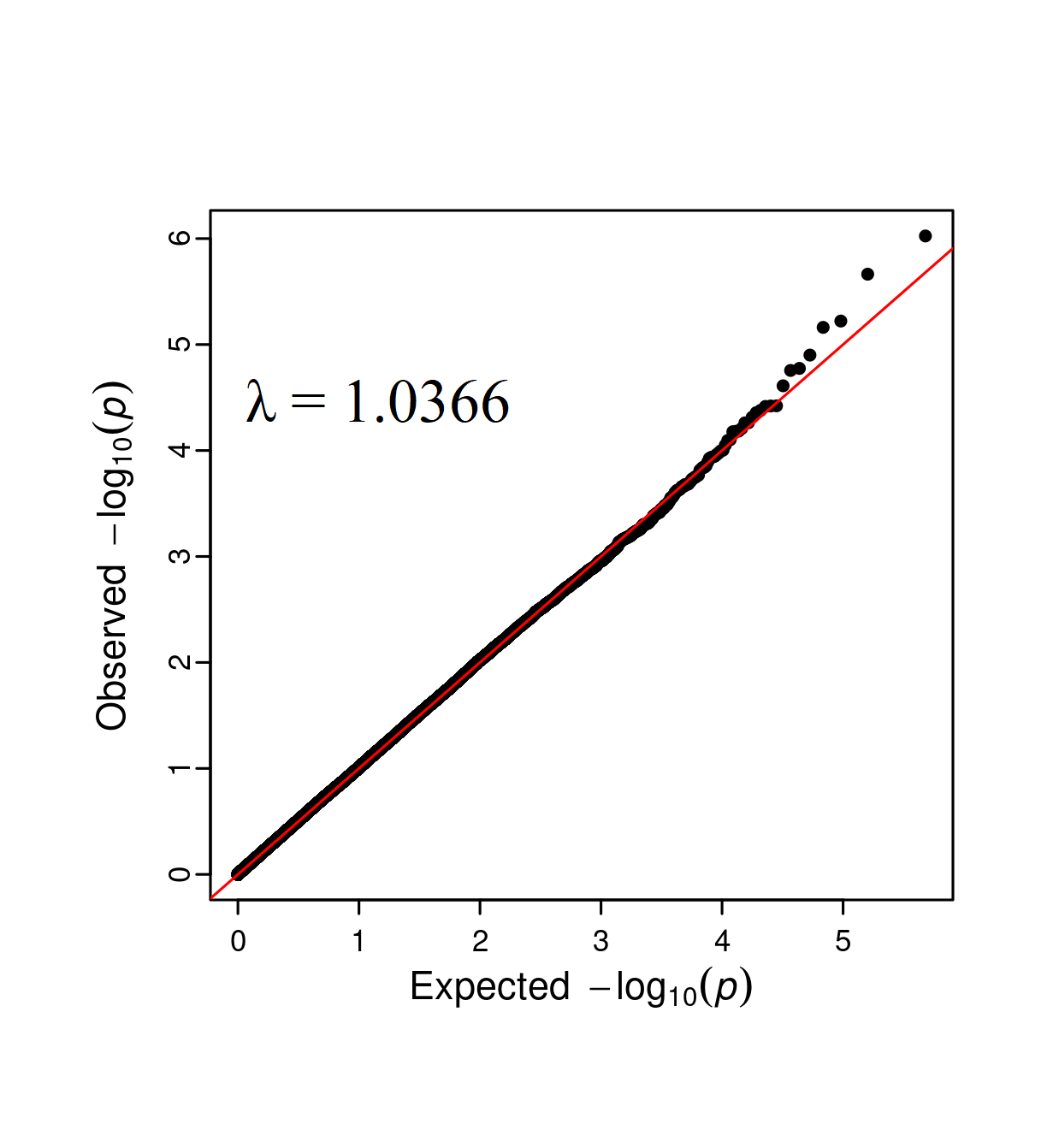


Figure S8. Q-Q plot and λ value of the genome-wide association analysis results for front leg view using high-density single nucleotide polymorphism genotypes in Canadian Holstein cattle.


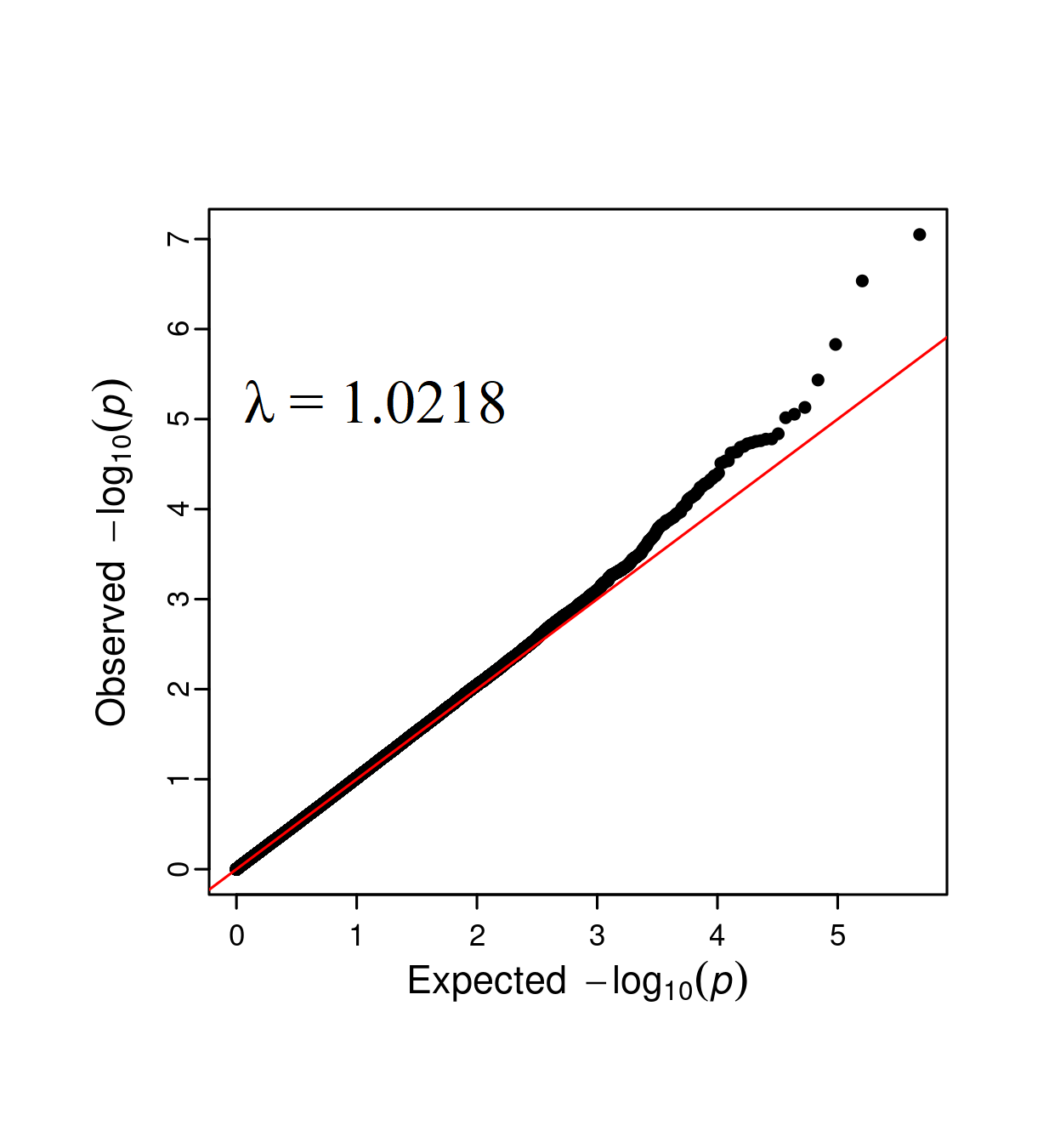


Figure S9. Q-Q plot and λ value of the genome-wide association analysis results for heel depth using high-density single nucleotide polymorphism genotypes in Canadian Holstein cattle.


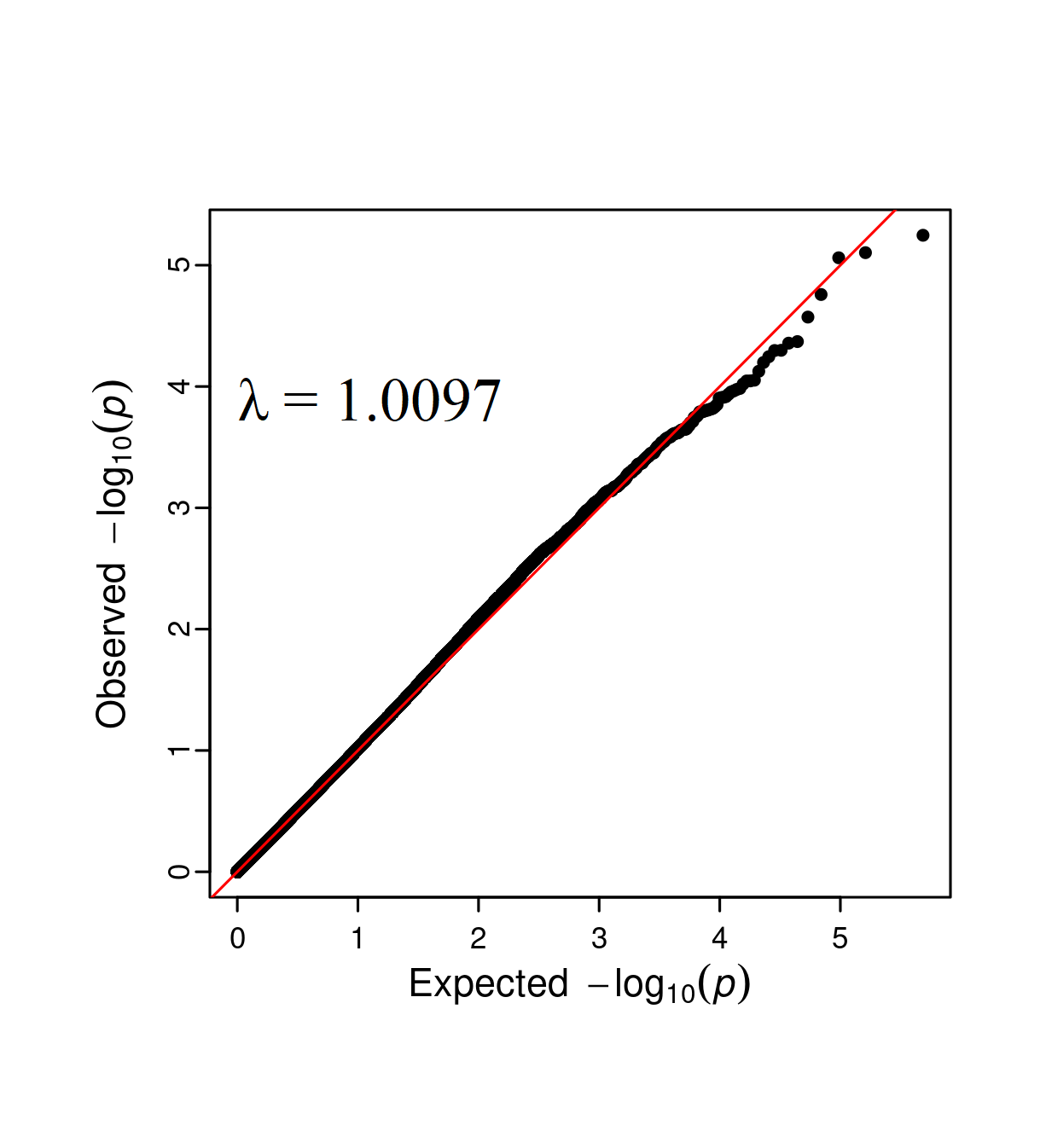


Figure S10. Q-plot and λ value of the genome-wide association analysis results for height at front end using high-density single nucleotide polymorphism genotypes in Canadian Holstein cattle.


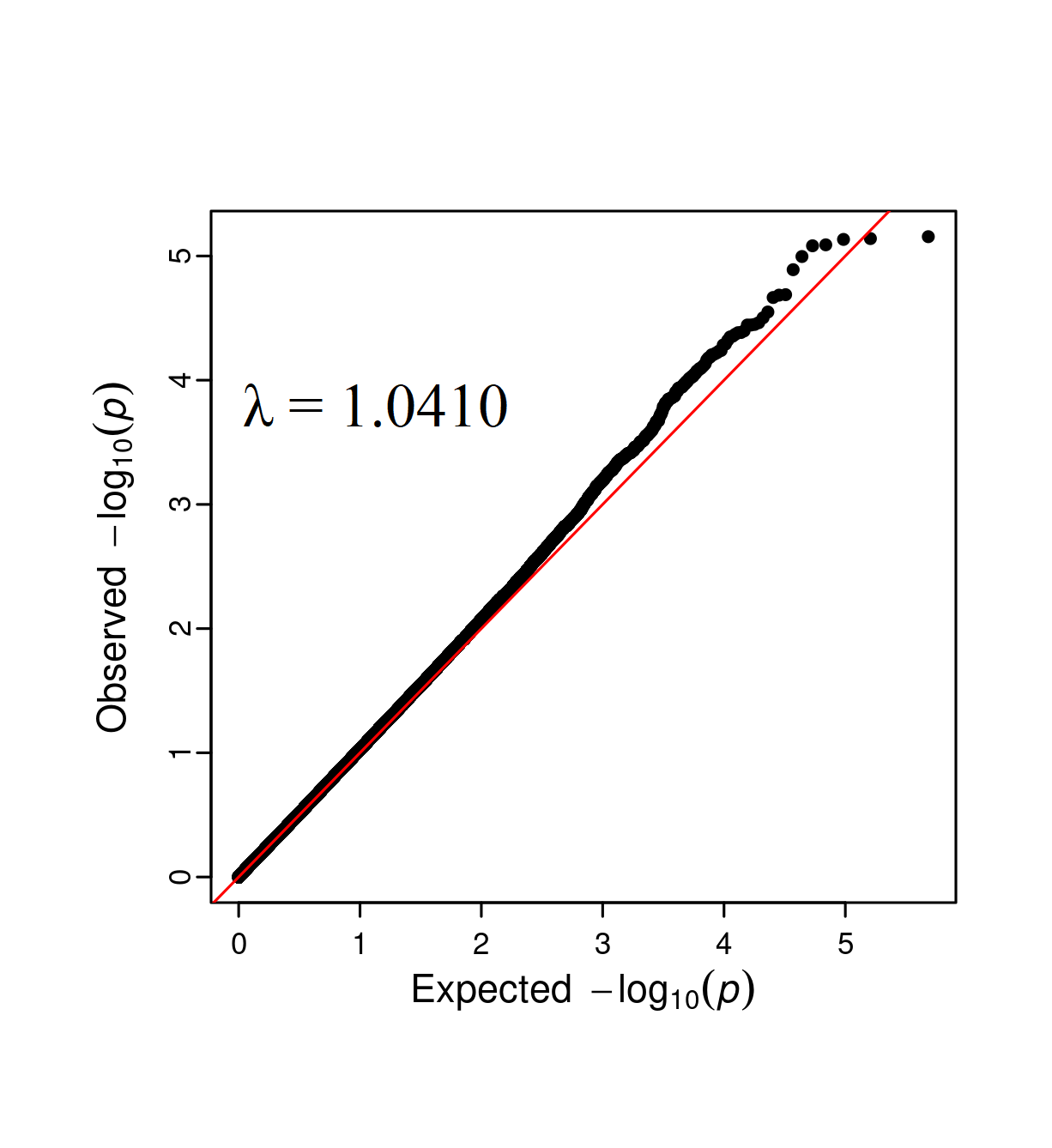


Figure S11. Q-Q plot and λ value of the genome-wide association analysis results for locomotion using high-density single nucleotide polymorphism genotypes in Canadian Holstein cattle.


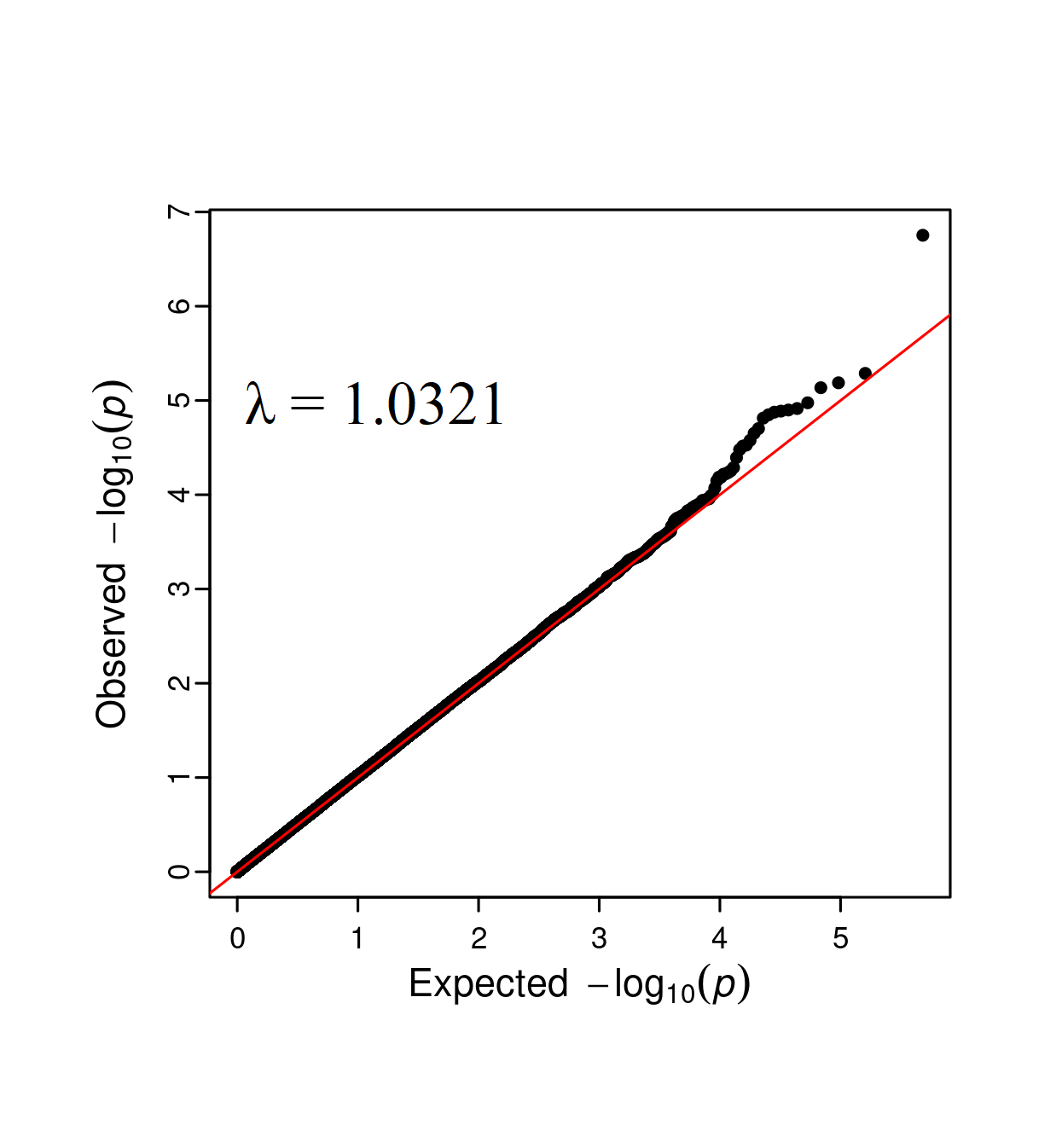


Figure S12. Q-Q plot and λ value of the genome-wide association analysis results for rear leg rearview using high-density single nucleotide polymorphism genotypes in Canadian Holstein cattle.


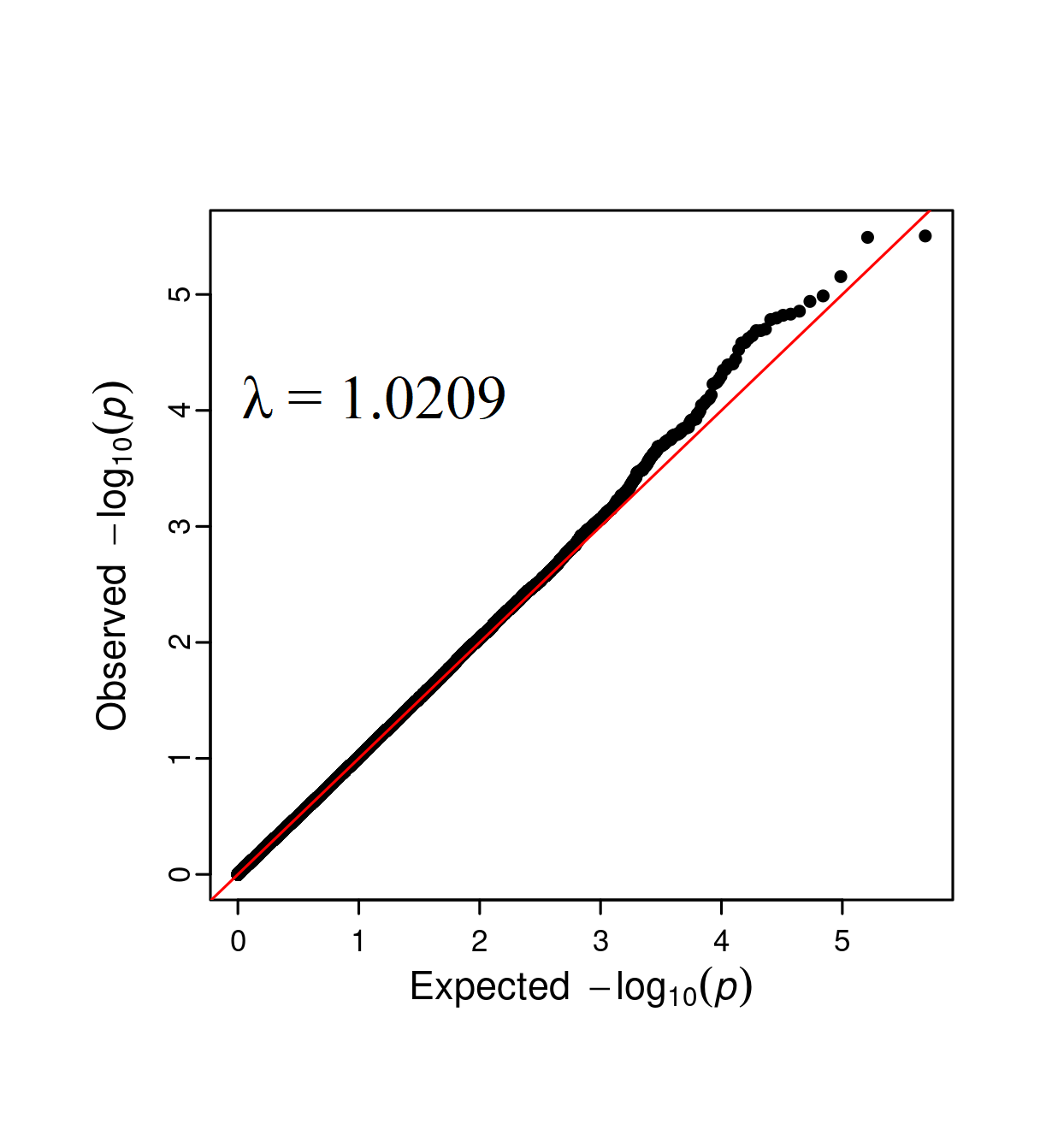


Figure S13. Q-Q plot and λ value of the genome-wide association analysis results for rear leg side view using high-density single nucleotide polymorphism genotypes in Canadian Holstein cattle.


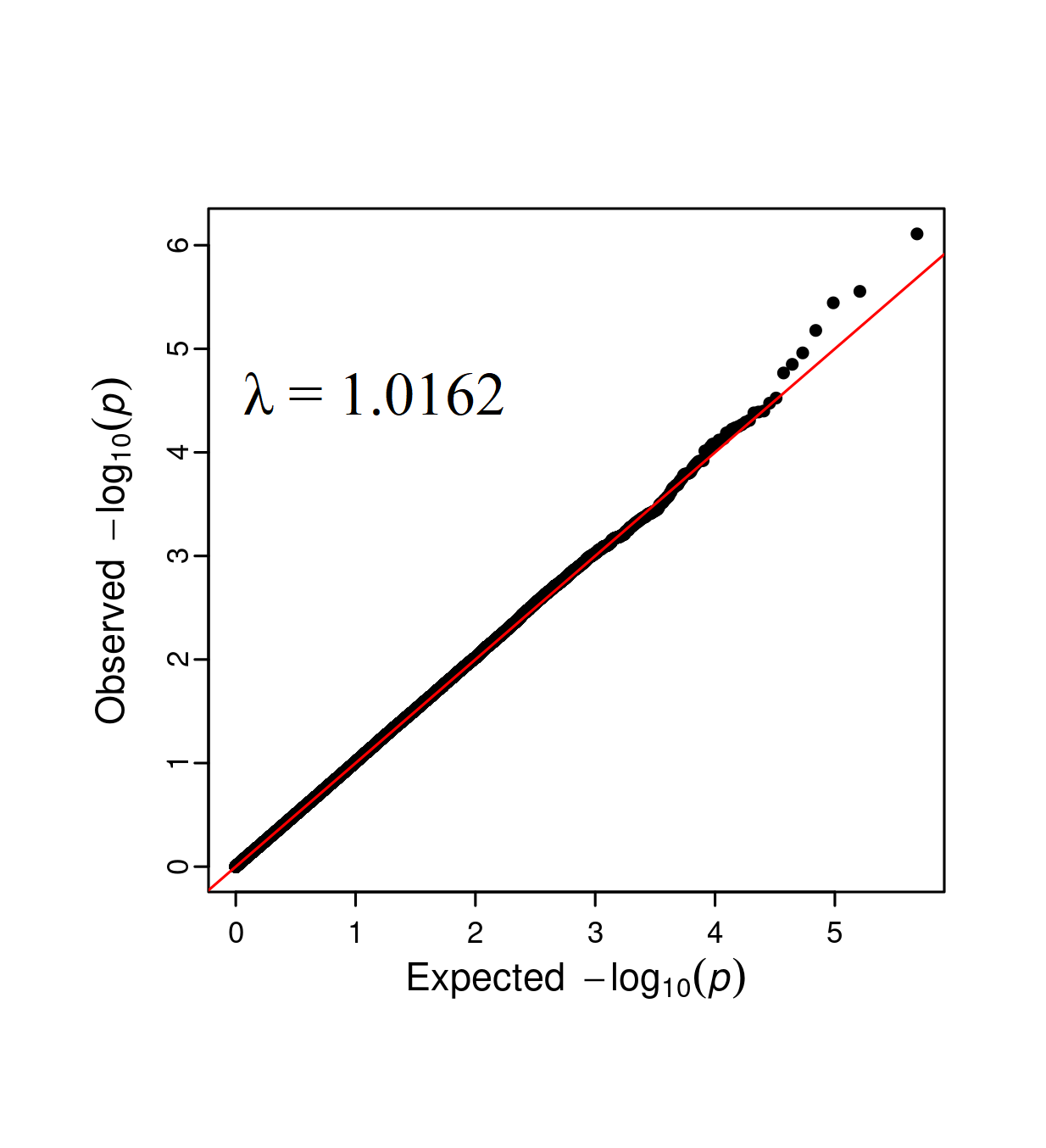


Figure S14. Q-Q plot and λ value of the genome-wide association analysis results for stature using high-density single nucleotide polymorphism genotypes in Canadian Holstein cattle.
