# Supplementary material for: Genome-wide association and functional genomic analyses for body conformation traits in North American Holstein cattle
Source: Front Genet. 2024 Oct 24;15:1478788. doi: 10.3389/fgene.2024.1478788 (PMC11540798; doi:10.3389/fgene.2024.1478788)
Supplement: Supplementary file 3 [file Presentation3.zip › Supplementary file 3.docx]

**SUPPLEMENTARY FIGURES AND TABLES**

**
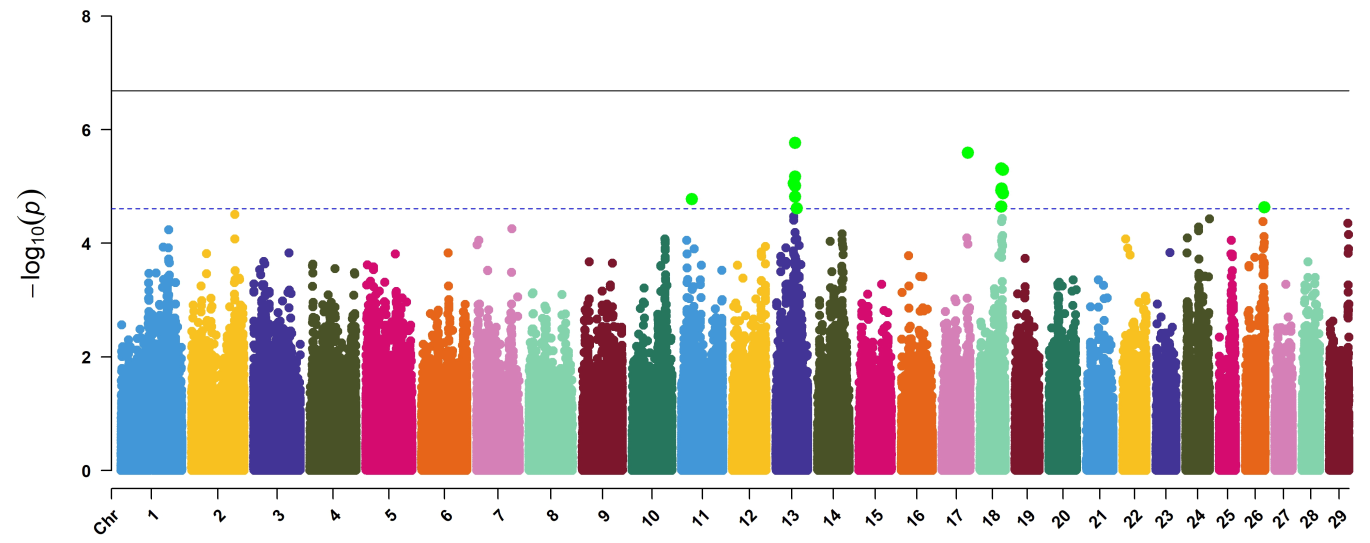
**

**Figure S1.** Manhattan plot of the genome-wide association analysis for composite Feet and Legs score index using imputed high-density single nucleotide polymorphism (SNP) panel data in Canadian Holstein cattle. The statistically significant SNPs after a genome-wide modified Bonferroni correction are colored in green, above the blue dotted line. The statistically significant SNPs after Bonferroni correction are colored in red, above the black line.

**
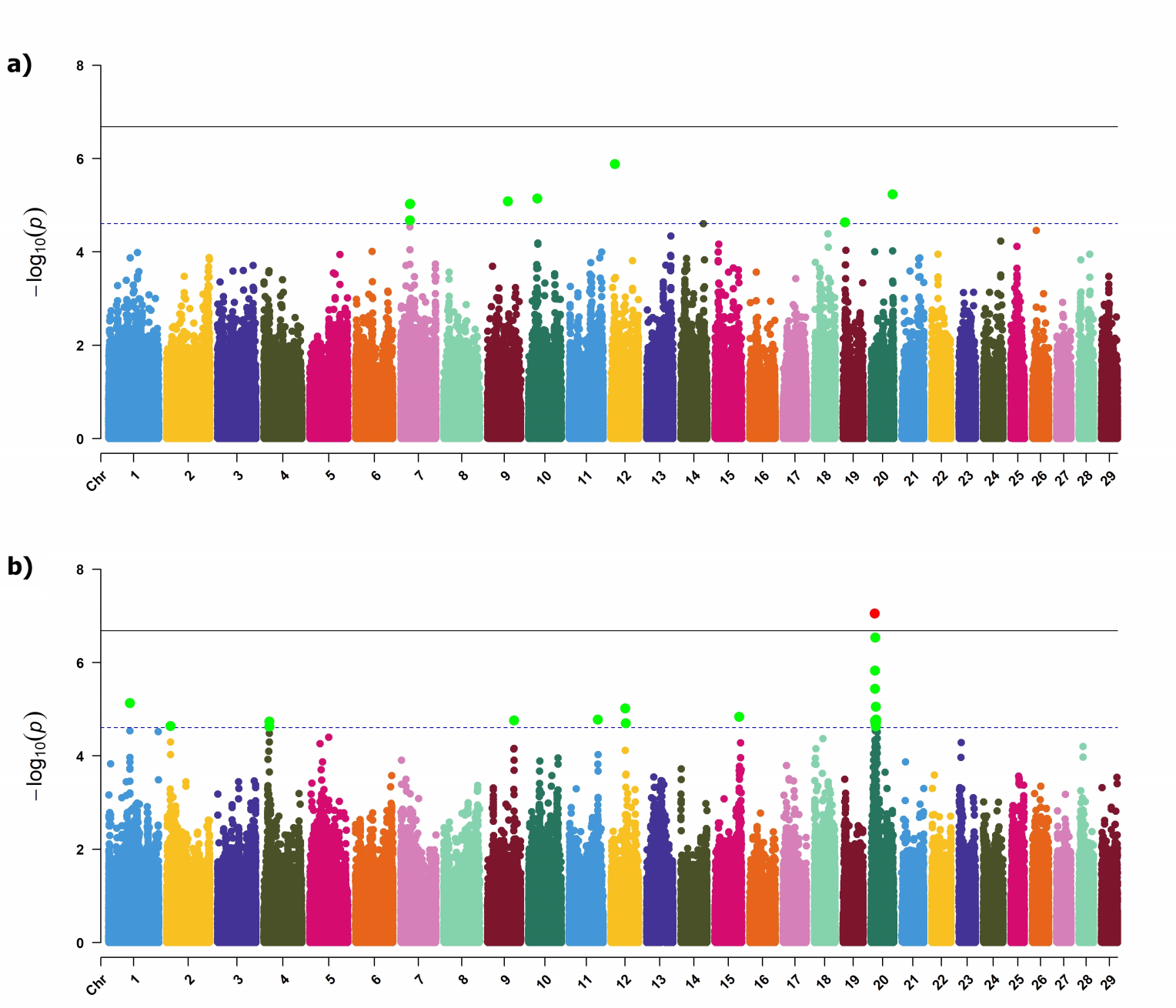
**

**Figure S2.** Manhattan plot of the genome-wide association analysis for foot angle (a) and heel depth (b) using imputed high-density single nucleotide polymorphism (SNP) panel data in Canadian Holstein cattle. The statistically significant SNPs after a genome-wide modified Bonferroni correction are colored in green, above the blue dotted line. The statistically significant SNPs after Bonferroni correction are colored in red, above the black line.

**
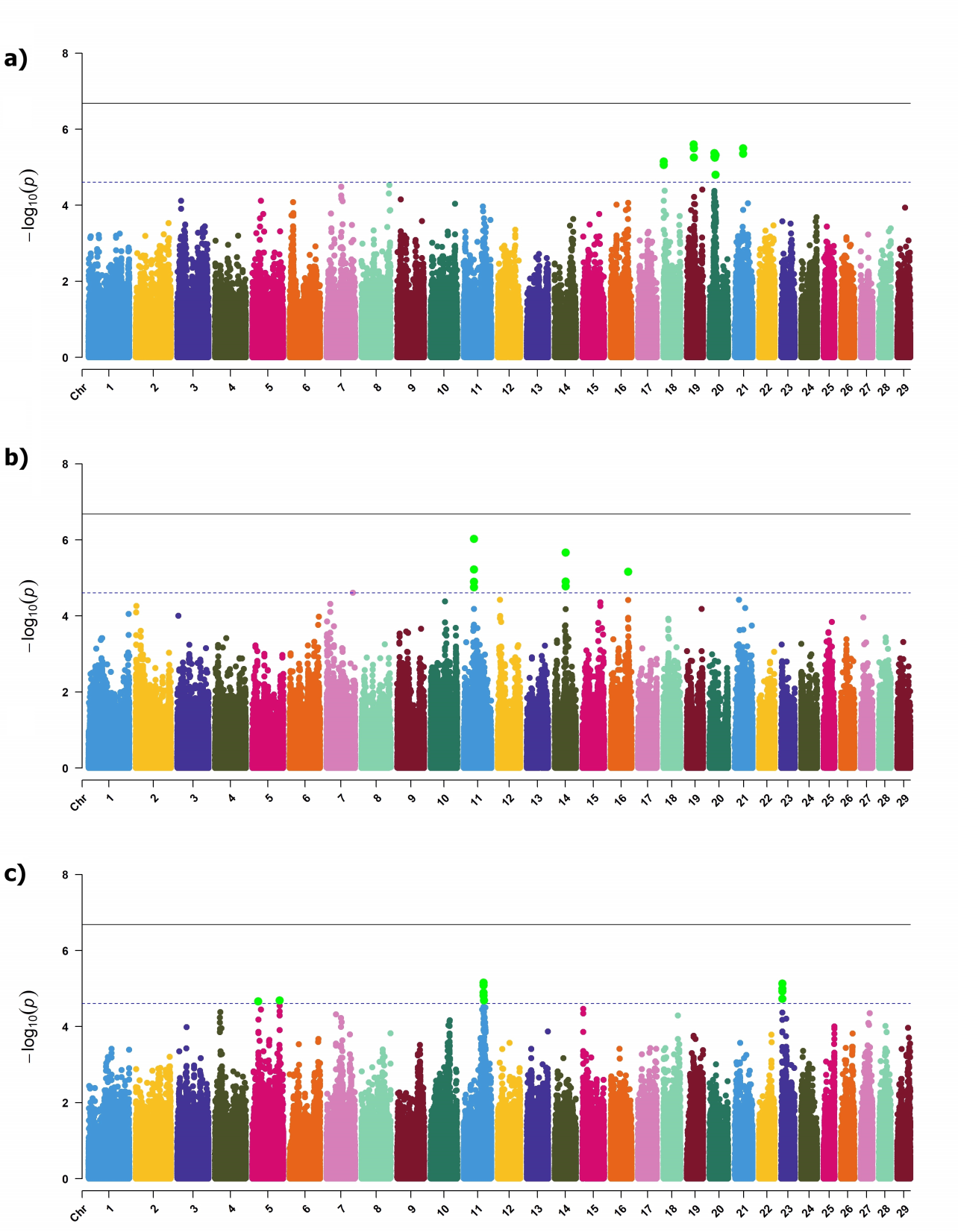
**

**Figure S3.** Manhattan plot of the genome-wide association analysis for bone quality (a), front leg view (b), and locomotion (c) using imputed high-density single nucleotide polymorphism (SNP) panel data in Canadian Holstein cattle. The statistically significant SNPs after a genome-wide modified Bonferroni correction are colored in green, above the blue dotted line. The statistically significant SNPs after Bonferroni correction are colored in red, above the black line.


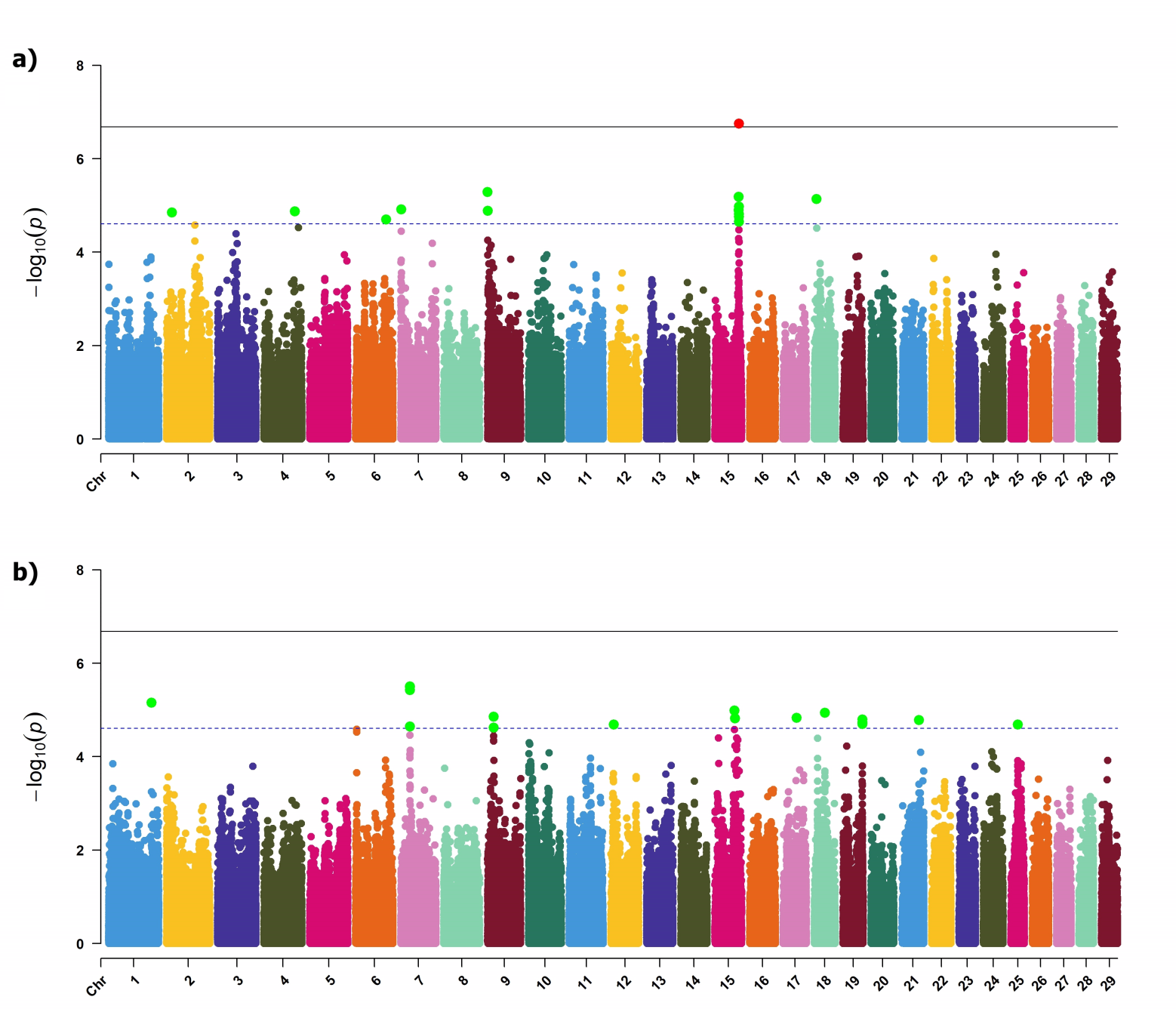


**Figure S4.** Manhattan plot of the genome-wide association analysis for rear leg rear view (a) and rear leg side view (b) using imputed high-density single nucleotide polymorphism (SNP) panel data in Canadian Holstein cattle. The statistically significant SNPs after a genome-wide modified Bonferroni correction are colored in green, above the blue dotted line. The statistically significant SNPs after Bonferroni correction are colored in red, above the black line.


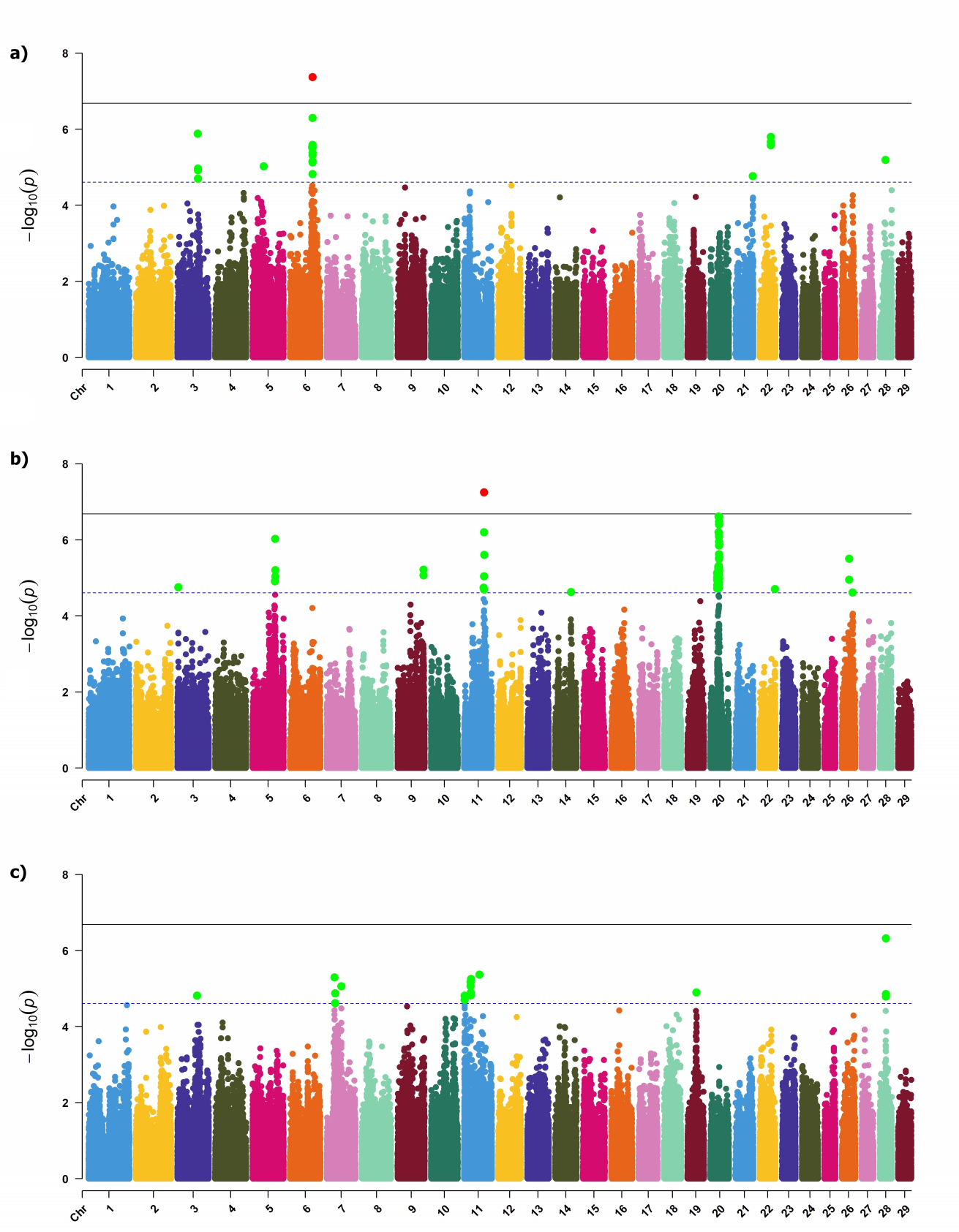


**Figure S5.** Manhattan plot of the genome-wide association analysis for body condition score (a), body depth (b), and chest width (c) using imputed high-density single nucleotide polymorphism (SNP) panel data in Canadian Holstein cattle. The statistically significant SNPs after a genome-wide modified Bonferroni correction are colored in green, above the blue dotted line. The statistically significant SNPs after Bonferroni correction are colored in red, above the black line.


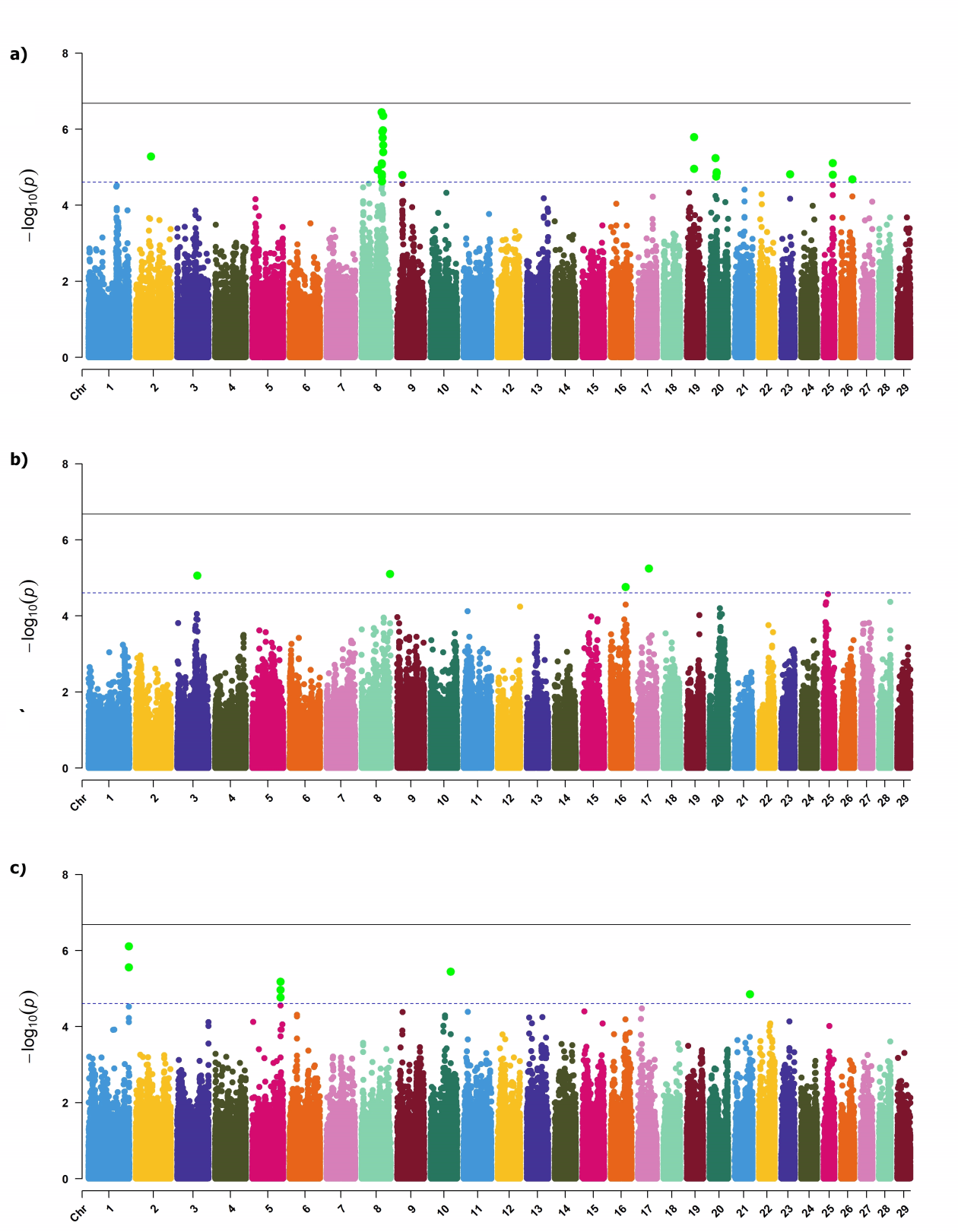


**Figure S6.** Manhattan plot of the genome-wide association analysis for dairy capacity (a), height at front end (b), and stature (c) using imputed high-density single nucleotide polymorphism (SNP) panel data in Canadian Holstein cattle. The statistically significant SNPs after a genome-wide modified Bonferroni correction are colored in green, above the blue dotted line. The statistically significant SNPs after Bonferroni correction are colored in red, above the black line.

**Table S1**. Single nucleotide polymorphisms (SNPs) associated with composite Feet and Legs score index and the corresponding positional genes identified.

| **Chr** | **Variant** | **Location (bp)** | **MAF** | **Effect** | **P-value** | **Genes** |
| --- | --- | --- | --- | --- | --- | --- |
| BTA11 | rs136174419* | 26,592,724 | 0.070, | 0.659 | 1.667 x 10^-05^ | *PPM1B* |
| BTA13 | rs41661000* | 50,453,781 | 0.496 | 0.471 | 1.713 x 10^-06^ | *ENSBTAG00000007199* |
|  | rs109648982* | 50,448,257 | 0.418 | 0.439 | 1.514 x 10^-05^ | *ENSBTAG00000007199* |
|  | rs134704848* | 50,459,617 | 0.303 | 0.451 | 9.787 x 10^-06^ | *ENSBTAG00000007199* |
|  | rs42280937* | 50,502,256 | 0.378 | 0.445 | 6.672 x 10^-06^ | *ENSBTAG00000007199, HAO1* |
|  | rs136408519* | 46,861,675 | 0.456 | -0.485 | 8.835 x 10^-06^ | *DIP2C, ZMYND11* |
|  | rs109244187* | 54,357,411 | 0.137 | 0.639 | 2.423 x 10^-05^ | *CHRNA4, COL20A1, ARFGAP1, ENSBTAG00000051598, BIRC7, YTHDF1, BHLHE23, SLC17A9* |
| BTA17 | rs41849069* | 67,138,540 | 0.218 | -0.539 | 2.543 x 10^-06^ | *MN1* |
| BTA18 | rs134739530* | 55,365,713 | 0.072 | -0.638 | 4.845 x 10^-06^ | *SPHK2, DBP, CA11, NTN5, SEC1, FUT2, MAMSTR, RASIP1, IZUMO1, FUT1, FGF21, BCAT2, HSD17B14, PLEKHA4* |
|  | rs133544315* | 59,727,750 | 0.124 | -0.574 | 5.095 x 10^-06^ | *-* |
|  | rs110774115* | 56,589,786 | 0.148 | 0.520 | 1.085 x 10^-05^ | *KCNC3, NAPSA, ENSBTAG00000048283, NR1H2, POLD1, SPIB, MYBPC2, GARIN5A, EMC10, JOSD2, LRRC4B* |
|  | rs134119868* | 55,513,275 | 0.033 | -0.772 | 2.257 x 10^-05^ | *BCAT2, HSD17B14, PLEKHA4, PPP1R15A, TULP2, NUCB1, DHDH, BAX, FTL, GYS1, RUVBL2, LHB, ENSBTAG00000052788, NTF4, ENSBTAG00000048800* |
| BTA 26 | rs3423553517 | 49,970,179 | 0.157 | 0.473 | 2.331 x 10^-05^ | *-* |

BTA – *Bos taurus* autosome; Chr.: chromosome; MAF: minor allele frequency; *Significant variant for Bonferroni correction based on independent chromosomal segments.

**Table S2.** Significant terms in the functional annotation analysis for group of genes identified for body condition score (BCS), bone quality (BQ), composite Feet & Legs score index (FL) and rear leg side view (RLSV) in Holstein cattle.

| **Trait** | **Category** | **Term** | **Genes** | FDR |
| --- | --- | --- | --- | --- |
| BCS | GOTERM_MF_DIRECT | Aryl sulfotransferase activity | [*SULT1B1, SULT1D1, SULT1E1*](https://david.ncifcrf.gov/geneReportFull.jsp?rowids=521920) | 4.0 x 10^-3^ |
| BCS | GOTERM_BP_DIRECT | Sulfation | [*SULT1B1, SULT1D1, SULT1E1*](https://david.ncifcrf.gov/geneReportFull.jsp?rowids=521920) | 5.2 x 10^-2^ |
| BCS | GOTERM_MF_DIRECT | sulfotransferase activity | [*SULT1B1, SULT1D1, SULT1E1*](https://david.ncifcrf.gov/geneReportFull.jsp?rowids=521920) | 2.9 x 10^-2^ |
| BCS | INTERPRO | Sulfotransferase domain | [*SULT1B1, SULT1D1, SULT1E1*](https://david.ncifcrf.gov/geneReportFull.jsp?rowids=521920) | 4.8 x 10^-2^ |
| BCS | UP_SEQ_FEATURE | DOMAIN:Sulfotransfer_1 | [*SULT1B1, SULT1D1, SULT1E1*](https://david.ncifcrf.gov/geneReportFull.jsp?rowids=521920) | 3.6 x 10^-2^ |
| BQ | UP_KW_DOMAIN | [Transmembrane helix](http://www.uniprot.org/keywords/?query=KW-1133) | *CTDNEP1, ELOVL7, CHRNB1, CLDN7, EFNB3, IL31RA, IL6ST, NLGN2, SLC2A4, SPEM1, TMEM102, TMEM256, TMEM88, TMEM95* | 4.2 x 10^-2^ |
| FL | INTERPRO | Glycosyl transferase, family 11 | *SEC1, FUT1, FUT2* | 1.2 x 10^-3^ |
| FL | GOTERM_MF_DIRECT | Galactoside 2-alpha-L-fucosyltransferase activity | *SEC1, FUT1, FUT2* | 2.4 x 10^-3^ |
| FL | GOTERM_MF_DIRECT | Alpha-(1,2)-fucosyltransferase activity | *SEC1, FUT1, FUT2* | 2.4 x 10^-3^ |
| RLSV | INTERPRO | [ABC transporter A, ABCA](https://www.ebi.ac.uk/interpro/entry/InterPro/IPR026082) | *ENSBTAG00000049286, ABCA10, ABCA5, ABCA6* | 2.9 x 10^-6^ |
| RLSV | INTERPRO | [ABC transporter-like](https://www.ebi.ac.uk/interpro/entry/InterPro/IPR003439) | *ABCA10, ABCA5, ABCA6* | 1.4 x 10^-2^ |
| RLSV | GOTERM_MF_DIRECT | [ATP binding](http://www.ebi.ac.uk/QuickGO/GTerm?id=GO:0005524) | *ABCA10, ABCA5, ABCA6, MAP2K6, PRKCB, RYK* | 3.5 x 10^-2^ |
| RLSV | UP_SEQ_FEATURE | DOMAIN:ABC transporter | *ABCA10, ABCA5, ABCA6* | 1.6 x 10^-2^ |

**Table S3**. Single nucleotide polymorphisms (SNPs) associated with foot angle (FAN) and heel depth (HDe) and the corresponding positional genes identified.

| **Trait** | **Chr** | **Variant** | **Location (bp)** | **MAF** | **Effect** | **P-value** | **Genes** |
| --- | --- | --- | --- | --- | --- | --- | --- |
| FAN | BTA7 | rs41628990* | 29,802,791 | 0.172 | 5.859 | 9.366 x 10^-06^ | *ENSBTAG00000046696* |
| FAN | BTA7 | rs42650571* | 28,300,663 | 0.291 | 4.825 | 9.435 x 10^-06^ | *-* |
| FAN | BTA7 | rs137103653* | 28,218,213 | 0.242 | -5.012 | 2.117 x 10^-05^ | *-* |
| FAN | BTA9 | rs136724399* | 64,526,199 | 0.032 | -12.006 | 8.232 x 10^-06^ | *-* |
| FAN | BTA10 | rs110339743* | 27,310,922 | 0.043 | 8.401 | 7.164 x 10^-06^ | *OR4N4, OR4N2, OR4K6, OR4K3, OR4K2, OR4K5, OR4K1, OR4K14, OR4K15, OR4Q2, ENSBTAG00000039370* |
| FAN | BTA12 | rs133014265* | 12,322,089 | 0.063 | -7.737 | 1.310 x 10^-06^ | *ENSBTAG00000053271, DGKH, AKAP11* |
| FAN | BTA19 | rs110348847* | 6,401,911 | 0.405 | -4.259 | 2.316 x 10^-05^ | *PCTP, ENSBTAG00000053407, ENSBTAG00000039563* |
| FAN | BTA20 | rs110955019* | 68,453,470 | 0.401 | 4.687 | 5.855 x 10^-06^ | *-* |
| HDe | BTA1 | rs41628909* | 67,619,208 | 0.335 | 0.691 | 7.418 x 10^-06^ | *SEC22A, ADCY5* |
| HDe | BTA2 | rs110383851* | 12,107,533 | 0.088 | 0.903 | 2.311 x 10^-05^ | *-* |
| HDe | BTA4 | rs135171504* | 17,221,951 | 0.276 | -0.645 | 1.830 x 10^-05^ | *-* |
| HDe | BTA4 | rs137817399* | 17,239,280 | 0.345 | -0.598 | 2.374 x 10^-05^ | *-* |
| HDe | BTA9 | rs109591644* | 83,309,979 | 0.120 | 0.852 | 1.737 x 10^-05^ | *SHPRH, GRM1* |
| HDe | BTA11 | rs110533337* | 90,106,355 | 0.227 | 0.641 | 1.661 x 10^-05^ | *RSAD2, CMPK2* |
| HDe | BTA12 | rs134493843* | 44,476,396 | 0.272 | 0.679 | 9.653 x 10^-06^ | *KLHL1* |
| HDe | BTA12 | rs43700792* | 47,035,822 | 0.364 | 0.614 | 2.005 x 10^-05^ | *-* |
| HDe | BTA15 | rs41781867* | 76,895,774 | 0.325 | 0.631 | 1.455 x 10^-05^ | *LRP4, CSTPP1* |
| HDe | BTA20 | rs41936372** | 11,927,432 | 0.469 | -0.714 | 8.917 x 10^-08^ | *-* |
| HDe | BTA20 | rs133321805* | 13,449,726 | 0.214 | 0.827 | 2.917 x 10^-07^ | *SREK1, ERBIN* |
| HDe | BTA20 | rs42243798* | 12,754,394 | 0.453 | 0.682 | 1.481 x 10^-06^ | *ENSBTAG00000049964* |
| HDe | BTA20 | rs132869581* | 12,784,933 | 0.327 | -0.717 | 3.677 x 10^-06^ | *ENSBTAG00000049964* |
| HDe | BTA20 | rs137824537* | 14,872,927 | 0.187 | 0.764 | 8.825 x 10^-06^ | *SHISAL2B, RGS7BP* |
| HDe | BTA20 | rs134796181* | 15,811,787 | 0.244 | 0.692 | 1.670 x 10^-05^ | *-* |
| HDe | BTA20 | rs110681916* | 12,095,813 | 0.413 | -0.603 | 1.765 x 10^-05^ | *-* |
| HDe | BTA20 | rs41658458* | 16,083,771 | 0.284 | 0.653 | 1.880 x 10^-05^ | *-* |
| HDe | BTA20 | rs109719590* | 13,994,440 | 0.179 | -0.753 | 2.058 x 10^-05^ | *SGTB, TRAPPC13, ENSBTAG00000047828, TRIM23, PPWD1, CENPK, ADAMTS6* |
| HDe | BTA20 | rs135322173* | 14,842,827 | 0.220 | 0.681 | 2.342 x 10^-05^ | *SREK1IP1, SHISAL2B, RGS7BP* |

BTA – *Bos taurus* autosome; Chr.: chromosome; MAF: minor allele frequency; *Significant variant for Bonferroni correction based on independent chromosomal segments; **Significant variant for Bonferroni multiple testing correction.

**Table S4.** Significant terms in the functional annotation analysis for group of genes identified for foot angle (FAN) in Holstein cattle.

| **Category** | **Term** | **Genes** | FDR |
| --- | --- | --- | --- |
| INTERPRO | Olfactory receptor | *ENSBTAG00000039370, OR4K1, OR4K15, OR4K14, OR4K2, OR4K3, OR4K5, OR4K6, OR4N2, OR4N4, OR4Q2* | 1.4 x 10^-8^ |
| GOTERM_MF_DIRECT | Olfactory receptor activity | *ENSBTAG00000039370, OR4K1, OR4K15, OR4K14, OR4K2, OR4K3, OR4K5, OR4K6, OR4N2, OR4N4, OR4Q2* | 6.9 x 10^-8^ |
| GOTERM_MF_DIRECT | G-protein coupled receptor activity | *ENSBTAG00000039370, OR4K1, OR4K15, OR4K14, OR4K2, OR4K3, OR4K5, OR4K6, OR4N2, OR4N4, OR4Q2* | 2.1 x 10^-7^ |
| KEGG_PATHWAY | Olfactory transduction | *OR4K1, OR4K15, OR4K14, OR4K2, OR4K3, OR4K5, OR4K6, OR4N2, OR4N4, OR4Q2* | 7.4 x 10^-7^ |
| UP_KW_BIOLOGICAL_PROCESS | Olfaction | *ENSBTAG00000039370, OR4K1, OR4K15, OR4K14, OR4K2, OR4K3, OR4K5, OR4K6, OR4N2, OR4Q2* | 1.8 x 10^-6^ |
| UP_KW_BIOLOGICAL_PROCESS | Sensory transduction | *ENSBTAG00000039370, OR4K1, OR4K15, OR4K14, OR4K2, OR4K3, OR4K5, OR4K6, OR4N2, OR4Q2* | 1.8 x 10^-6^ |
| UP_SEQ_FEATURE | DOMAIN:G_PROTEIN_RECEP_F1_2 | *ENSBTAG00000039370, OR4K1, OR4K15, OR4K14, OR4K2, OR4K3, OR4K5, OR4K6, OR4N2, OR4N4, OR4Q2* | 2.4 x 10^-4^ |
| INTERPRO | G protein-coupled receptor, rhodopsin-like | *ENSBTAG00000039370, OR4K1, OR4K15, OR4K14, OR4K2, OR4K3, OR4K5, OR4K6, OR4N2, OR4N4, OR4Q2* | 1.0 x 10^-4^ |
| INTERPRO | GPCR, rhodopsin-like, 7TM | *ENSBTAG00000039370, OR4K1, OR4K15, OR4K14, OR4K2, OR4K3, OR4K5, OR4K6, OR4N2, OR4N4, OR4Q2* | 1.0 x 10^-4^ |
| GOTERM_CC_DIRECT | Plasma membrane | *ENSBTAG00000039370, OR4K1, OR4K15, OR4K14, OR4K2, OR4K3, OR4K5, OR4K6, OR4N2, AKAP11, OR4Q2* | 2.0 x 10^-4^ |
| UP_KW_CELLULAR_COMPONENT | Cell membrane | *ENSBTAG00000039370, OR4K1, OR4K15, OR4K14, OR4K2, OR4K3, OR4K5, OR4K6, OR4N2, OR4Q2* | 5.3 x 10^-4^ |
| GOTERM_CC_DIRECT | Integral component of membrane | *ENSBTAG00000039370, OR4K1, OR4K15, OR4K14, OR4K2, OR4K3, OR4K5, OR4K6, OR4N2, OR4N4, OR4Q2* | 2.1 x 10^-3^ |
| UP_KW_MOLECULAR_FUNCTION | Transducer | *ENSBTAG00000039370, OR4K1, OR4K15, OR4K14, OR4K2, OR4K3, OR4K5, OR4K6, OR4N2, OR4N4, OR4Q2* | 4.7 x 10^-3^ |
| UP_KW_MOLECULAR_FUNCTION | Receptor | *ENSBTAG00000039370, OR4K1, OR4K15, OR4K14, OR4K2, OR4K3, OR4K5, OR4K6, OR4N2, OR4N4, OR4Q2* | 6.7 x 10^-3^ |

**Table S5**. Single nucleotide polymorphisms (SNPs) associated with bone quality (BQ), front leg view (FLV) and locomotion (LOC) and the corresponding positional genes identified.

| **Trait** | **Chr** | **Variant** | **Location (bp)** | **MAF** | **Effect** | **P-value** | **Genes** |
| --- | --- | --- | --- | --- | --- | --- | --- |
| BQ | BTA18 | rs109766155* | 1,498,235 | 0.204 | -0.700 | 7.133 x 10^-06^ | *VAC14, MTSS2, ENSBTAG00000050915, IL34, SF3B3* |
| BQ | BTA18 | rs136556784* | 1,470,422 | 0.208 | -0.692 | 8.702 x 10^-06^ | *VAC14, MTSS2, ENSBTAG00000050915, IL34* |
| BQ | BTA19 | rs136174626* | 27,056,493 | 0.219 | -0.925 | 2.530 x 10^-06^ | *CTDNEP1, ELP5, CLDN7, SLC2A4, YBX2, EIF5A, GPS2, NEURL4, ENSBTAG00000045892, KCTD11, TMEM95, TNK1, PLSCR3, TMEM256, NLGN2, SPEM1, SPEM2, TMEM102, ENSBTAG00000050569, CHRNB1, ZBTB4, POLR2A* |
| BQ | BTA19 | rs110845473* | 27,484,633 | 0.215 | -0.898 | 5.533 x 10^-06^ | *TP53, WRAP53, EFNB3, DNAH2, KDM6B, TMEM88, NAA38, CYB5D1, CHD3* |
| BQ | BTA20 | HAPMAP50241-BTA-115966* | 18,571,733 | 0.145 | -0.868 | 4.235 x 10^-06^ | *ELOVL7, ENSBTAG00000049955, DEPDC1B* |
| BQ | BTA20 | rs41613300* | 18,626,250 | 0.145 | -0.868 | 4.235 x 10^-06^ | *ELOVL7, DEPDC1B* |
| BQ | BTA20 | rs110877560* | 23,265,588 | 0.275 | -0.718 | 4.725 x 10^-06^ | *IL6ST, IL31RA, DDX4* |
| BQ | BTA20 | rs108968345* | 23,282,550 | 0.303 | -0.697 | 4.916 x 10^-06^ | *IL6ST, IL31RA, DDX4* |
| BQ | BTA20 | rs132818385* | 18,725,426 | 0.357 | -0.678 | 5.355 x 10^-06^ | *DEPDC1B* |
| BQ | BTA20 | rs110558195* | 20,142,479 | 0.159 | 0.832 | 5.468 x 10^-06^ | *PDE4D* |
| BQ | BTA20 | rs109445617* | 23,266,165 | 0.186 | -0.746 | 1.589 x 10^-05^ | *IL6ST, IL31RA, DDX4* |
| BQ | BTA20 | rs29011426* | 23,312,762 | 0.186 | -0.746 | 1.589 x 10^-05^ | *IL6ST, IL31RA, DDX4* |
| BQ | BTA21 | ARS-BFGL-NGS-35280* | 33,069,961 | 0.169 | -0.852 | 3.170 x 10^-06^ | *ENSBTAG00000024311, ODF3L1* |
| FLV | BTA11 | rs136468307* | 39,657,165 | 0.079 | -12.325 | 9.448 x 10^-07^ | *-* |
| FLV | BTA11 | rs133253380* | 40,113,682 | 0.127 | -6.870 | 6.001 x 10^-06^ | *-* |
| FLV | BTA11 | rs109196408* | 39,491,318 | 0.083 | -10.081 | 1.755 x 10^-05^ | *-* |
| FLV | BTA14 | rs134079793* | 42,586,262 | 0.222 | -4.609 | 2.167 x 10^-06^ | *-* |
| FLV | BTA14 | rs109291207* | 42,898,907 | 0.457 | -4.089 | 1.256 x 10^-05^ | *HEY1, STMN2, ENSBTAG00000032812* |
| FLV | BTA14 | rs110192073* | 42,975,630 | 0.460 | -4.035 | 1.678 x 10^-05^ | *ENSBTAG00000032812, HEY1* |
| FLV | BTA16 | rs109182378* | 67,865,555 | 0.446 | 4.034 | 6.886 x 10^-06^ | *PLA2G4A* |
| LOC | BTA5 | rs41654529* | 105,743,393 | 0.085 | -0.721 | 2.066 x 10^-05^ | *FGF6, FGF23, TIGAR, CCND2,* |
| LOC | BTA5 | rs109678909* | 22,971,796 | 0.308 | -0.449 | 2.158 x 10^-05^ | *-* |
| LOC | BTA11 | rs137570291* | 76,829,758 | 0.493 | 0.512 | 6.991 x 10^-06^ | *-* |
| LOC | BTA11 | rs41612857* | 76,863,768 | 0.490 | -0.515 | 7.239 x 10^-06^ | *-* |
| LOC | BTA11 | rs110778731* | 76,836,424 | 0.476 | -0.511 | 8.128 x 10^-06^ | *-* |
| LOC | BTA11 | rs29019720* | 76,779,895 | 0.374 | -0.468 | 8.276 x 10^-06^ | *-* |
| LOC | BTA11 | rs136286714* | 76,930,841 | 0.479 | -0.500 | 1.289 x 10^-05^ | *-* |
| LOC | BTA11 | rs109011936* | 78,444,403 | 0.384 | 0.560 | 2.048 x 10^-05^ | *ENSBTAG00000049117, PUM2* |
| LOC | BTA23 | rs136696041* | 5,075,497 | 0.158 | 0.562 | 7.355 x 10^-06^ | *HMGCLL1, GFRAL, HCRTR2* |
| LOC | BTA23 | rs109057916* | 5,081,980 | 0.177 | 0.521 | 1.009 x 10^-05^ | *HMGCLL1, GFRAL, HCRTR2* |

BTA – *Bos taurus* autosome; Chr.: chromosome; MAF: minor allele frequency; *Significant variant for Bonferroni correction based on independent chromosomal segments

**Table S6**. Single nucleotide polymorphisms (SNPs) associated with rear leg rear view (RLRV) and rear leg side view (RLSV) and the corresponding positional genes identified.

| **Trait** | **Chr** | **Variant** | **Location (bp)** | **MAF** | **Effect** | **P-value** | **Genes** |
| --- | --- | --- | --- | --- | --- | --- | --- |
| RLRV | BTA2 | BOVINEHD0200004707* | 16,529,717 | 0.102 | -7.930 | 1.419 x 10^-05^ | *-* |
| RLRV | BTA4 | rs134960239* | 97,420,163 | 0.033 | 14.864 | 1.331 x 10^-05^ | *EXOC4* |
| RLRV | BTA6 | rs109648337* | 96,284,651 | 0.317 | 5.268 | 1.986 x 10^-05^ | *RASGEF1B, ENSBTAG00000052070* |
| RLRV | BTA7 | rs41658116* | 1,114,190 | 0.473 | 4.838 | 1.217 x 10^-05^ | *RASGEF1C, RNF130* |
| RLRV | BTA9 | rs3423094135* | 64,186 | 0.297 | -5.379 | 5.159 x 10^-06^ | *-* |
| RLRV | BTA9 | rs43744542* | 414,987 | 0.223 | -5.678 | 1.297 x 10^-05^ | *PTP4A1* |
| RLRV | BTA15 | rs41781092** | 75,567,845 | 0.285 | -6.427 | 1.767 x 10^-07^ | *ENSBTAG00000054083* |
| RLRV | BTA15 | rs42912957* | 75,357,973 | 0.079 | 9.559 | 6.492 x 10^-06^ | *PRDM11, SYT13* |
| RLRV | BTA15 | rs110279884* | 76,060,265 | 0.441 | 5.235 | 1.058 x 10^-05^ | *PHF21A* |
| RLRV | BTA15 | rs110287097* | 75,439,705 | 0.157 | 6.926 | 1.262 x 10^-05^ | *-* |
| RLRV | BTA15 | rs109713642* | 75,887,663 | 0.500 | -5.076 | 1.538 x 10^-05^ | *MAPK8IP1, C15H11orf94, PEX16, LARGE2, PHF21A, SLC35C1, CRY2* |
| RLRV | BTA15 | rs109435828* | 76,007,949 | 0.496 | 4.967 | 2.227 x 10^-05^ | *MAPK8IP1, C15H11orf94, PEX16, LARGE2, PHF21A, CRY2* |
| RLRV | BTA18 | rs109828867* | 7,150,509 | 0.013 | 17.809 | 7.323 x 10^-06^ | *-* |
| RLSV | BTA1 | rs43270148* | 135,158,833 | 0.017 | 12.253 | 7.018 x 10^-06^ | *RYK* |
| RLSV | BTA7 | rs3423241779* | 28,761,924 | 0.171 | 4.058 | 3.132 x 10^-06^ | *-* |
| RLSV | BTA7 | rs132945415* | 28,662,752 | 0.825 | -4.050 | 3.219 x 10^-06^ | *-* |
| RLSV | BTA7 | rs3423227599* | 28,744,865 | 0.151 | 3.835 | 2.267 x 10^-05^ | *-* |
| RLSV | BTA9 | rs136977277* | 19,418,690 | 0.254 | -3.646 | 1.394 x 10^-05^ | *SH3BGRL2* |
| RLSV | BTA9 | rs29013705* | 19,421,211 | 0.229 | -3.643 | 2.393 x 10^-05^ | *SH3BGRL2* |
| RLSV | BTA12 | rs41627857* | 8,747,286 | 0.363 | -3.267 | 2.049 x 10^-05^ | *-* |
| RLSV | BTA15 | rs136483399* | 61,666,789 | 0.258 | 3.552 | 1.029 x 10^-05^ | *DCDC1* |
| RLSV | BTA15 | rs42625101* | 63,705,526 | 0.052 | 6.150 | 1.512 x 10^-05^ | *QSER1, DEPDC7, TCP11L1, CSTF3* |
| RLSV | BTA17 | rs110240722* | 42,379,700 | 0.050 | 9.195 | 1.478 x 10^-05^ | *PDGFC* |
| RLSV | BTA18 | rs136114993* | 33,182,132 | 0.191 | -4.135 | 1.148 x 10^-05^ | *-* |
| RLSV | BTA19 | rs134677011* | 61,146,690 | 0.479 | 3.235 | 1.597 x 10^-05^ | *MAP2K6* |
| RLSV | BTA19 | rs3423448968* | 61,353,943 | 0.445 | 3.267 | 1.990 x 10^-05^ | *ABCA5, ENSBTAG00000049286, ABCA10, ABCA6* |
| RLSV | BTA21 | rs110016525* | 54,528,550 | 0.271 | 3.505 | 1.645 x 10^-05^ | *ENSBTAG00000052854* |
| RLSV | BTA25 | rs42684377* | 21,882,759 | 0.290 | 4.062 | 2.051 x 10^-05^ | *PRKCB, CACNG3* |

BTA – *Bos taurus* autosome; Chr.: chromosome; MAF: minor allele frequency; *Significant variant for Bonferroni correction based on independent chromosomal segments

**Table S7**. Single nucleotide polymorphisms (SNPs) associated with body condition score (BCS) and chest width (CW) and the corresponding positional genes identified.

| **Trait** | **Chr** | **Variant** | **Location (bp)** | **MAF** | **Effect** | **P-value** | **Genes** |
| --- | --- | --- | --- | --- | --- | --- | --- |
| BCS | BTA3 | rs110919821* | 78,508,362 | 0.082 | -1.661 | 1.318 x 10^-06^ | *MIER1, DNAI4, ENSBTAG00000049696, INSL5, DYNLT5, SGIP1* |
| BCS | BTA3 | rs3423155914* | 79,371,143 | 0.061 | -1.889 | 1.090 x 10^-05^ | *PDE4B, MGC137454* |
| BCS | BTA3 | rs110146466* | 79,405,288 | 0.063 | -1.883 | 1.195 x 10^-05^ | *PDE4B, MGC137454* |
| BCS | BTA3 | rs42318770* | 79,398,869 | 0.058 | 1.970 | 2.000 x 10^-05^ | *PDE4B, MGC137454* |
| BCS | BTA5 | rs43440584* | 42,830,228 | 0.377 | 0.798 | 9.539 x 10^-06^ | *PTPRB, PTPRR* |
| BCS | BTA6 | rs110434046** | 87,184,768 | 0.410 | -0.987 | 4.262 x 10^-08^ | *NPFFR2* |
| BCS | BTA6 | rs109452259* | 87,068,809 | 0.470 | -0.895 | 5.100 x 10^-07^ | *GC, ENSBTAG00000049290* |
| BCS | BTA6 | rs134055603* | 87,097,860 | 0.470 | -0.895 | 5.100 x 10^-07^ | *GC* |
| BCS | BTA6 | rs110310151* | 86,996,470 | 0.484 | -0.826 | 2.604 x 10^-06^ | *GC, ENSBTAG00000049290* |
| BCS | BTA6 | rs110694875* | 87,405,290 | 0.491 | -0.842 | 2.996 x 10^-06^ | *NPFFR2, ADAMTS3* |
| BCS | BTA6 | rs135052123* | 85,312,679 | 0.305 | -0.881 | 3.010 x 10^-06^ | *SULT1B1, SULT1D1, SULT1E1, CSN1S1* |
| BCS | BTA6 | rs110352004* | 87,213,962 | 0.488 | -0.810 | 4.223 x 10^-06^ | *NPFFR2* |
| BCS | BTA6 | rs109547247* | 87,000,075 | 0.484 | -0.774 | 6.977 x 10^-06^ | *GC, ENSBTAG00000049290* |
| BCS | BTA6 | rs109420430* | 87,409,266 | 0.441 | -0.820 | 7.494 x 10^-06^ | *NPFFR2, ADAMTS3* |
| BCS | BTA6 | rs42766480* | 87,156,735 | 0.426 | -0.812 | 1.510 x 10^-05^ | *NPFFR2* |
| BCS | BTA21 | rs109750686* | 67,469,826 | 0.225 | 0.866 | 1.720 x 10^-05^ | *RCOR1, ENSBTAG00000046633, TRAF3* |
| BCS | BTA22 | rs134857381* | 42,618,135 | 0.016 | 2.578 | 1.595 x 10^-06^ | *ENSBTAG00000020373, FAM3D* |
| BCS | BTA22 | rs133236828* | 42,482,809 | 0.016 | 2.553 | 2.176 x 10^-06^ | *ENSBTAG00000020373* |
| BCS | BTA22 | rs109833935* | 42,628,033 | 0.015 | 2.541 | 2.654 x 10^-06^ | *ENSBTAG00000020373, FAM3D, FAM107A* |
| BCS | BTA28 | rs133217149* | 21,988,457 | -1.213 | 0.085 | 6.482 x 10^-06^ | *-* |
| CW | BTA3 | rs41616993* | 75,767,267 | 0.325 | 0.564 | 1.545 x 10^-05^ | *-* |
| CW | BTA7 | rs3423241949* | 29,402,654 | 0.173 | 0.793 | 5.060 x 10^-06^ | *-* |
| CW | BTA7 | rs41256154* | 32,035,639 | 0.170 | 0.641 | 1.345 x 10^-05^ | *FTMT* |
| CW | BTA7 | rs133650669* | 32,232,465 | 0.271 | 0.582 | 2.426 x 10^-05^ | *ENSBTAG00000054810, ENSBTAG00000003086* |
| CW | BTA7 | rs42284427* | 56,306,169 | 0.369 | -0.760 | 8.735 x 10^-06^ | *-* |
| CW | BTA11 | rs43615003* | 2,006,676 | 0.096 | 0.830 | 1.541 x 10^-05^ | *ENSBTAG00000034657, MRPS5, ZNF514, ENSBTAG00000048358, PROM2, KCNIP3* |
| CW | BTA11 | rs110125096* | 2,124,396 | 0.096 | 0.8168 | 2.012 x 10^-05^ | *PROM2, KCNIP3, FAHD2A, GPAT2* |
| CW | BTA11 | rs42848657* | 24,408,850 | 0.038 | 1.257 | 6.833 x 10^-06^ | *PKDCC* |
| CW | BTA11 | rs109414585* | 25,163,431 | 0.032 | 1.382 | 8.637 x 10^-06^ | *MTA3, OXER1, HAAO* |
| CW | BTA11 | rs137112475* | 25,540,060 | 0.033 | 1.294 | 1.514 x 10^-05^ | *ZFP36L2, THADA* |
| CW | BTA11 | rs43671061* | 25,616,082 | 0.039 | -1.280 | 1.283 x 10^-05^ | *THADA* |
| CW | BTA11 | rs29018553* | 26,317,389 | 0.032 | 1.410 | 5.618 x 10^-06^ | *-* |
| CW | BTA11 | rs42948529* | 59,551,844 | 0.294 | -0.655 | 4.306 x 10^-06^ | *-* |
| CW | BTA19 | rs41911826* | 34,164,616 | 0.350 | 0.567 | 1.275 x 10^-05^ | *B9D1, EPN2, GRAP, SLC5A10, FAM83G, RNF112, MFAP4, MAPK7* |
| CW | BTA28 | rs136922313* | 23,410,406 | 0.182 | 0.648 | 1.403 x 10^-05^ | *CTNNA3, LRRTM3* |
| CW | BTA28 | rs42139508* | 23,422,787 | 0.375 | 0.586 | 4.802 x 10^-07^ | *CTNNA3, LRRTM3* |
| CW | BTA28 | rs137289883* | 23,730,009 | 0.315 | 0.509 | 1.634 x 10^-05^ | *-* |

BTA – *Bos taurus* autosome; Chr.: chromosome; MAF: minor allele frequency; *Significant variant for Bonferroni correction based on independent chromosomal segments; **Significant variant for Bonferroni multiple testing correction.

**Table S8**. Single nucleotide polymorphisms (SNPs) associated with body depth (BD) and the corresponding positional genes identified.

| **Trait** | **Chr** | **Variant** | **Location (bp)** | **MAF** | **Effect** | **P-value** | **Genes** |
| --- | --- | --- | --- | --- | --- | --- | --- |
| BD | BTA3 | rs109285537* | 3,248,489 | 0.173 | -0.678 | 1.767 x 10^-05^ | *TMCO1, ALDH9A1, MGST3* |
| BD | BTA5 | rs135033432* | 86,668,039 | 0.132 | 1.040 | 1.233 x 10^-05^ | *SOX5* |
| BD | BTA5 | rs135250417* | 87,757,852 | 0.408 | 0.588 | 9.405 x 10^-07^ | *C2CD5* |
| BD | BTA5 | rs109795387* | 88,379,767 | 0.464 | -0.540 | 6.275 x 10^-06^ | *ABCC9, KCNJ8* |
| BD | BTA5 | rs136530412* | 88,687,403 | 0.460 | -0.545 | 9.266 x 10^-06^ | *GYS2, SPX, GOLT1B, RECQL, PYROXD1* |
| BD | BTA9 | rs133168118* | 100,204,200 | 0.076 | -0.868 | 6.116 x 10^-06^ | *-* |
| BD | BTA9 | rs136337205* | 100,567,862 | 0.133 | 0.729 | 8.558 x 10^-06^ | *PDE10A* |
| BD | BTA11 | rs109018444* | 75,301,517 | 0.335 | 0.592 | 1.825 x 10^-05^ | *ATAD2B, KLHL29* |
| BD | BTA11 | rs133999160* | 76,738,472 | 0.421 | -0.568 | 2.027 x 10^-05^ | *-* |
| BD | BTA11 | rs137570291* | 76,829,758 | 0.492 | 0.621 | 9.135 x 10^-06^ | *-* |
| BD | BTA11 | rs108938667** | 76,875,084 | 0.486 | -0.789 | 5.640 x 10^-08^ | *-* |
| BD | BTA11 | rs136262208* | 76,910,156 | 0.156 | -0.748 | 2.006 x 10^-05^ | *-* |
| BD | BTA11 | rs110632976* | 76,915,355 | 0.493 | -0.717 | 6.301 x 10^-07^ | *-* |
| BD | BTA11 | rs3423322590* | 77,740,182 | 0.412 | -0.635 | 2.467 x 10^-06^ | *TDRD15* |
| BD | BTA14 | rs134380044* | 61,489,677 | 0.202 | -0.640 | 2.347 x 10^-05^ | *ATP6V1C1, AZIN1* |
| BD | BTA20 | rs110622993* | 26,819,224 | 0.097 | -0.799 | 1.888 x 10^-05^ | *-* |
| BD | BTA20 | rs109281540* | 26,918,633 | 0.074 | -1.088 | 1.155 x 10^-05^ | *-* |
| BD | BTA20 | rs41601571* | 26,929,602 | 0.162 | -1.103 | 1.013 x 10^-05^ | *-* |
| BD | BTA20 | rs108943316* | 27,015,667 | 0.158 | 0.974 | 1.553 x 10^-05^ | *-* |
| BD | BTA20 | rs110731830* | 27,035,603 | 0.111 | -1.011 | 7.378 x 10^-06^ | *-* |
| BD | BTA20 | rs109101658* | 27,067,049 | 0.159 | -0.771 | 9.783 x 10^-06^ | *-* |
| BD | BTA20 | rs135547893* | 27,091,598 | 0.104 | 0.968 | 7.668 x 10^-06^ | *-* |
| BD | BTA20 | rs135634336* | 27,116,615 | 0.106 | -1.057 | 7.531 x 10^-06^ | *-* |
| BD | BTA20 | rs41567173* | 31,034,452 | 0.162 | 0.829 | 5.478 x 10^-06^ | *ENSBTAG00000033187* |
| BD | BTA20 | rs29022980* | 31,069,095 | 0.163 | 0.824 | 5.695 x 10^-06^ | *ENSBTAG00000033187, NNT* |
| BD | BTA20 | rs134893516* | 31,094,282 | 0.164 | 0.832 | 4.995 x 10^-06^ | *ENSBTAG00000033187, NNT* |
| BD | BTA20 | rs29018641* | 31,134,096 | 0.162 | 0.829 | 5.491 x 10^-06^ | *ENSBTAG00000033187, NNT* |
| BD | BTA20 | rs41580285* | 31,177,589 | 0.199 | 0.737 | 9.948 x 10^-06^ | *ENSBTAG00000033187, NNT, PAIP1* |
| BD | BTA20 | rs41639261* | 31,912,365 | 0.242 | -0.799 | 2.430 x 10^-07^ | *GHR* |
| BD | BTA20 | rs110482506* | 32,009,781 | 0.243 | -0.771 | 6.295 x 10^-07^ | *GHR* |
| BD | BTA20 | rs136824395* | 32,478,922 | 0.293 | 0.820 | 1.855 x 10^-05^ | *-* |
| BD | BTA20 | rs135891055* | 32,629,298 | 0.202 | -0.703 | 1.093 x 10^-05^ | *FBXO4, RIMOC1, OXCT1* |
| BD | BTA20 | rs42376179* | 33,256,142 | 0.240 | -0.668 | 1.485 x 10^-05^ | *PLCXD3, C6* |
| BD | BTA20 | rs137300246* | 33,275,529 | 0.163 | -0.849 | 4.815 x 10^-06^ | *PLCXD3, C6* |
| BD | BTA20 | rs110708973* | 33,312,140 | 0.148 | -0.905 | 2.376 x 10^-06^ | *PLCXD3, C6* |
| BD | BTA20 | rs134597209* | 33,314,180 | 0.165 | 0.902 | 1.129 x 10^-06^ | *PLCXD3, C6* |
| BD | BTA20 | rs43001858* | 33,323,766 | 0.193 | -0.774 | 8.432 x 10^-06^ | *C6* |
| BD | BTA20 | rs41938455* | 33,341,878 | 0.118 | -1.103 | 3.307 x 10^-07^ | *C6* |
| BD | BTA20 | rs110600376* | 33,381,855 | 0.193 | 0.820 | 2.995 x 10^-06^ | *C6, MROH2B* |
| BD | BTA20 | rs41938115* | 33,392,509 | 0.148 | 0.968 | 6.561 x 10^-07^ | *C6, MROH2B* |
| BD | BTA20 | rs41948991* | 33,429,456 | 0.176 | -0.809 | 3.204 x 10^-06^ | *C6, MROH2B* |
| BD | BTA20 | rs110772002* | 33,537,716 | 0.166 | -0.920 | 8.314 x 10^-07^ | *C6, MROH2B, C7, CARD6* |
| BD | BTA20 | rs136028188* | 33,541,612 | 0.189 | -0.852 | 1.400 x 10^-06^ | *C6, MROH2B, C7, CARD6* |
| BD | BTA20 | rs135466944* | 33,614,197 | 0.142 | -1.011 | 3.942 x 10^-07^ | *MROH2B, C7, CARD6, RPL37, PRKAA1, TTC33* |
| BD | BTA20 | rs134892111* | 33,634,634 | 0.164 | -0.769 | 1.763 x 10^-05^ | *C7, CARD6, RPL37, PRKAA1, TTC33* |
| BD | BTA20 | rs41944921* | 33,748,963 | 0.213 | 0.709 | 8.778 x 10^-06^ | *RPL37, PRKAA1, TTC33, PTGER4* |
| BD | BTA20 | rs132687807* | 33,796,269 | 0.127 | -1.057 | 7.468 x 10^-07^ | *PRKAA1, TTC33, PTGER4* |
| BD | BTA20 | rs137330216* | 33,886,801 | 0.149 | 0.771 | 5.606 x 10^-06^ | *-* |
| BD | BTA20 | rs135463978* | 34,147,545 | 0.183 | -0.703 | 1.310 x 10^-05^ | *-* |
| BD | BTA20 | rs110120167* | 34,163,269 | 0.179 | -0.847 | 8.216 x 10^-07^ | *-* |
| BD | BTA20 | rs134098007* | 34,187,922 | 0.129 | -0.767 | 2.552 x 10^-07^ | *-* |
| BD | BTA20 | rs41939690* | 34,224,609 | 0.152 | -0.668 | 3.856 x 10^-07^ | *-* |
| BD | BTA20 | rs3423475152* | 34,256,033 | 0.130 | -0.675 | 1.350 x 10^-06^ | *-* |
| BD | BTA20 | rs41640156* | 34,460,986 | 0.181 | -0.769 | 6.572 x 10^-06^ | *-* |
| BD | BTA20 | rs43762676* | 34,507,773 | 0.189 | 0.645 | 1.040 x 10^-05^ | *-* |
| BD | BTA20 | rs29013890* | 34,800,041 | 0.138 | -0.768 | 2.887 x 10^-06^ | *-* |
| BD | BTA22 | rs110475161* | 58,788,655 | 0.154 | 0.644 | 1.971 x 10^-05^ | *IQSEC1* |
| BD | BTA26 | rs137381370* | 28,713,815 | 0.041 | 1.775 | 3.155 x 10^-06^ | *-* |
| BD | BTA26 | rs137837731* | 28,776,880 | 0.038 | 1.824 | 1.112 x 10^-05^ | *-* |
| BD | BTA26 | rs137141124* | 42,096,145 | 0.457 | -0.559 | 2.428 x 10^-05^ | *TACC2, BTBD16* |

BTA – *Bos taurus* autosome; Chr.: chromosome; MAF: minor allele frequency; *Significant variant for Bonferroni correction based on independent chromosomal segments; **Significant variant for Bonferroni multiple testing correction.

**Table S9**. Single nucleotide polymorphisms (SNPs) associated with dairy capacity (DC), height at front end (HFE) and stature (ST) and the corresponding positional genes identified.

| **Trait** | **Chr** | **Variant** | **Location (bp)** | **MAF** | **Effect** | **P-value** | **Genes** |
| --- | --- | --- | --- | --- | --- | --- | --- |
| DC | BTA2 | rs42530075* | 59,249,406 | 0.011 | -2.276 | 5.248 x 10^-06^ | *HNMT* |
| DC | BTA2 | rs42530109* | 59,353,958 | 0.012 | 2.276 | 5.248 x 10^-06^ | *ENSBTAG00000039437* |
| DC | BTA8 | rs43559726* | 62,766,388 | 0.170 | -0.871 | 1.180 x 10^-05^ | *TDRD7, TMOD1, TSTD2, NCBP1* |
| DC | BTA8 | rs43571286* | 78,118,013 | 0.137 | 1.140 | 3.566 x 10^-07^ | *NTRK2* |
| DC | BTA8 | rs41590554* | 78,350,218 | 0.171 | 0.918 | 1.770 x 10^-05^ | *NTRK2* |
| DC | BTA8 | rs134395443* | 78,566,341 | 0.170 | 0.998 | 7.943 x 10^-06^ | *-* |
| DC | BTA8 | rs109853697* | 78,583,825 | 0.212 | 0.887 | 1.516 x 10^-05^ | *ENSBTAG00000050767* |
| DC | BTA8 | rs110135009* | 78,585,174 | 0.498 | -0.708 | 1.596 x 10^-05^ | *ENSBTAG00000050767* |
| DC | BTA8 | rs42710122* | 78,587,452 | 0.258 | 0.841 | 8.545 x 10^-06^ | *ENSBTAG00000050767* |
| DC | BTA8 | rs42360833* | 79,729,695 | 0.258 | 0.783 | 2.344 x 10^-05^ | *-* |
| DC | BTA8 | rs134625094* | 80,674,372 | 0.127 | 1.172 | 1.164 x 10^-06^ | *DAPK1* |
| DC | BTA8 | rs43734710* | 82,161,159 | 0.144 | 1.079 | 1.675 x 10^-06^ | *PTCH1* |
| DC | BTA8 | rs137341434* | 82,787,089 | 0.129 | 1.149 | 1.065 x 10^-06^ | *ERCC6L2, ENSBTAG00000053599* |
| DC | BTA8 | rs43568478* | 83,673,324 | 0.151 | -1.039 | 4.041 x 10^-06^ | *ENSBTAG00000009764* |
| DC | BTA8 | rs43568461* | 83,687,485 | 0.185 | -0.901 | 2.623 x 10^-06^ | *ENSBTAG00000009764* |
| DC | BTA8 | rs110495184* | 83,707,862 | 0.126 | 1.237 | 4.450 x 10^-07^ | *ENSBTAG00000009764, ZNF484* |
| DC | BTA9 | rs110091464* | 20,235,961 | 0.214 | -0.860 | 1.595 x 10^-05^ | *-* |
| DC | BTA19 | rs137606216* | 28,575,558 | 0.212 | 0.905 | 1.612 x 10^-06^ | *NTN1, STX8* |
| DC | BTA19 | rs110053512* | 28,598,941 | 0.266 | 0.773 | 1.099 x 10^-05^ | *NTN1, STX8* |
| DC | BTA20 | rs137289069* | 23,431,592 | 0.242 | -0.812 | 5.788 x 10^-06^ | *IL31RA, DDX4, SLC38A9* |
| DC | BTA20 | rs110475392* | 26,060,070 | 0.324 | -0.754 | 1.756 x 10^-05^ | *MOCS2, ITGA2, ITGA1* |
| DC | BTA20 | rs111000237* | 27,202,535 | 0.241 | -0.799 | 1.456 x 10^-05^ | *-* |
| DC | BTA20 | rs137532092* | 27,329,790 | 0.281 | 0.780 | 1.358 x 10^-05^ | *-* |
| DC | BTA23 | rs137079734* | 34,479,993 | 0.193 | -0.850 | 1.539 x 10^-05^ | *ENSBTAG00000038430* |
| DC | BTA25 | rs42067830* | 35,354,001 | 0.171 | 0.795 | 7.849 x 10^-06^ | *COL26A1, IFT22, FIS1* |
| DC | BTA25 | rs42066764* | 35,399,705 | 0.243 | -0.718 | 1.574 x 10^-05^ | *COL26A1, IFT22, FIS1, CLDN15, ZNHIT1, PLOD3* |
| DC | BTA26 | rs135535498* | 44,187,259 | 0.493 | 0.624 | 2.094 x 10^-05^ | *LHPP, FAM53B* |
| HFE | BTA3 | rs43345563* | 77,599,781 | 0.281 | -2.114 | 8.686 x 10^-06^ | *GNG12, GADD45A* |
| HFE | BTA8 | rs43581101* | 110,185,962 | 0.034 | -5.065 | 7.883 x 10^-06^ | *MEGF9, FBXW2, B3GALT9, PSMD5, CUTAL, PHF19* |
| HFE | BTA16 | rs42464469* | 57,526,937 | 0.324 | 1.720 | 1.744 x 10^-05^ | *-* |
| HFE | BTA17 | rs137254844* | 42,276,117 | 0.025 | -4.772 | 5.667 x 10^-06^ | *PDGFC* |
| ST | BTA1 | BOVINEHD0100039562* | 156,730,566 | 0.048 | 10.368 | 7.770 x 10^-07^ | *KCNH8* |
| ST | BTA1 | rs42948748* | 156,744,423 | 0.035 | -10.847 | 2.789 x 10^-06^ | *KCNH8* |
| ST | BTA5 | rs136729009* | 109,344,409 | 0.160 | -5.695 | 1.711 x 10^-05^ | *MICAL3, PEX26, TUBA8, CDC42EP1, LGALS2* |
| ST | BTA5 | rs133183974* | 109,392,964 | 0.043 | -12.514 | 1.095 x 10^-05^ | *PEX26, TUBA8, CDC42EP1, LGALS2, GGA1, SH3BP1* |
| ST | BTA5 | rs110365530* | 109,615,284 | 0.086 | 6.906 | 6.646 x 10^-06^ | *LGALS1, NOL12, TRIOBP, ENSBTAG00000048772, GCAT, GALR3, ANKRD54, EIF3L, MICALL1* |
| ST | BTA10 | rs109396576* | 77,708,611 | 0.058 | 8.819 | 3.597 x 10^-06^ | *FUT8* |
| ST | BTA21 | rs110111160* | 60,066,050 | 0.224 | -4.869 | 1.412 x 10^-05^ | *DICER1, CLMN* |

BTA – *Bos taurus* autosome; Chr.: chromosome; MAF: minor allele frequency; *Significant variant for Bonferroni correction based on independent chromosomal segments
